# Supplementary material for: Effects of interferential stimulation on clinical symptom and urodynamic findings in women with voiding dysfunction: A protocol of randomized clinical trial
Source: PLoS One. 2025 Oct 27;20(10):e0330610. doi: 10.1371/journal.pone.0330610 (PMC12558555; doi:10.1371/journal.pone.0330610)
Supplement: S1 Checklist — (DOCX) [file pone.0330610.s001.docx]

| **Section/item** | **Address** |
| --- | --- |
| **Title** | **Title Page** |
| **Trial registration** | **Page 3** |
| **Funding** | **---** |
| **Roles and responsibilities** | **---** |
| **Background and rationale** | **Pages 4-7** |
| **Objectives** | **Page 7** |
| **Trial design** | **Page 7-8** |
| **Study setting** | **Page 8** |
| **Eligibility criteria** | **Pages 9** |
| **Interventions** | **Pages 14-17** |
| **Outcomes** | **Pages 9-14** |
| **Participant timeline** | **Page 8** |
| **Sample size** | **Page 19-20** |
| **Recruitment** | **Page 8** |
| **Allocation** | **Page 18** |
| **Blinding (masking)** | **Page 18-19** |
| **Data collection methods** | **Page 8** |
| **Data management** | **---** |
| **Statistical methods** | **Page 20-21** |
| **Data monitoring** | **Page 24** |
| **Harms** | **Page 24** |
| **Research ethics approval** | **Page 8** |
| **Protocol amendments** | **---** |
| **Consent or assent** | **Page 8** |
| **Confidentiality** | **---** |
| **Declaration of interests** | **---** |
| **Access to data** | **---** |

**SPIRIT 2013 Checklist.**
