# Supplementary material for: Effects of interferential stimulation on clinical symptom and urodynamic findings in women with voiding dysfunction: A protocol of randomized clinical trial
Source: PLoS One. 2025 Oct 27;20(10):e0330610. doi: 10.1371/journal.pone.0330610 (PMC12558555; doi:10.1371/journal.pone.0330610)
Supplement: S1 File — English Translate. (PDF) [file pone.0330610.s002.pdf]

project title

Investigating the effects of interferential stimulation on clinical symptom and urodynamic findings in women with voiding dysfunction: A randomized clinical trial

Tracking Code: 28251
Researcher: Seyedeh Saeedeh Babazadeh
Specialty: Physiotherapy
Initial Registration Date: January 29, 2024 14:23:09
Submission Date: March 6, 2024 22:26:53
Current Revision Date: August 26, 2024 09:54:24
Primary Review Center/Faculty: Iran University of Medical Sciences
Secondary Target Center: Faculty of Rehabilitation Sciences

General Project Specifications and Abstract

Grant Type:
First Submitted Proposal Grant

Field:
Other

Priority:
Other

Is this an animal study?
No

Keywords:

functional voiding dysfunction, interferential electrical stimulation, interferential stimulation, urodynamic, uroflowmetry, women

Persian Title:
بررسی تاثیر جریان اینترفرنشیال بر علائم بالینی و یافته های یورودینامیک در زنان مبتلا به اختلال تخلیه ادرار: یک کارآزمایی بالینی

Is this project a clinical trial?

Yes

University social responsibility

No

English Title

Investigating the effects of interferential stimulation on clinical symptom and urodynamic findings in women with voiding dysfunction: A randomized clinical trial

Selected Keywords

Research Line

Pelvic Floor Physiotherapy

Keywords

Voiding Dysfunction, Functional voiding Dysfunction, Interferential Electrical Current, Interferential stimulation, Urodynamics, Uroflowmetry, Women

---

Type of project

Practical research

Introduction-Problem Statement

Introduction

Incomplete bladder emptying is a prevalent clinical issue in women, associated with impaired bladder voiding (1). Due to variations in methodology and definitions across studies, there is no precise estimation of its prevalence. However, urodynamic findings suggest that up to 2% of adults are affected by this condition (2). The prevalence of voiding dysfunction in women ranges from 6.8% to 61.7%, depending on diagnostic criteria, and increases with age (3). This disorder encompasses symptoms experienced during or after voiding, with the most frequently reported being a sensation of incomplete emptying, followed by weak urinary stream. In some cases, it may coexist with storage-phase symptoms. In these cases the underlying pathology may be either independent or interrelated (1, 2, 4).

Normal micturition begins with a voluntary reduction in intraurethral pressure due to relaxation of the urethra and surrounding striated muscles. This is followed by sustained contraction of the detrusor muscle, leading to complete bladder emptying within a defined period. Thus, normal voiding is characterized by an unobstructed, rapid flow with minimal post-void residual (PVR) volume, achieved through urethral relaxation without elevated detrusor pressure (2).

The primary causes of voiding dysfunction are either impaired detrusor contractility or bladder outlet obstruction (BOO) (1). A prospective study found that most cases

are attributed to BOO (2). BOO is defined as increased detrusor pressure and decreased urinary flow rate during voiding, in the absence of infection or obvious injury. It may be associated with elevated PVR (2, 5). In women, BOO arises from structural or functional etiologies. The main functional causes are dysfunctional voiding (DV) and detrusor-sphincter dyssynergia (DSD) (1).

According to the International Continence Society, DV is characterized by intermittent or fluctuating urinary flow due to involuntary contractions of the periurethral striated muscles during voiding in neurologically intact individuals. DSD, as a neurological disorder, involves involuntary coincidence of urethral or periurethral striated muscle contractions with detrusor contraction. This sometimes leading to complete urinary interruption (5, 6). DSD is observed in patients with spinal cord injuries affecting the brainstem and sacral spinal cord relations (7).

Problem Statement

BOO is typically diagnosed via pressure-flow studies assessing urinary flow rate and detrusor pressure (2). In women, a bladder outlet obstruction index (BOOI) >18 indicates obstructive voiding dysfunction. Elevated PVR (>50 mL) and excessive daytime urinary frequency are key evaluable symptoms (8, 9). Patients often face challenges such as frequent bathroom visits, sleep disruption, and persistent daytime anxiety (10). Chronic or acute urinary retention (painful or painless) and urinary tract infections are additional complications (1).

Definitive diagnosis of voiding dysfunction relies on patient history and urodynamic testing (1). A 3-day voiding diary, uroflowmetry, ultrasound-determined PVR, pelvic floor electromyography (EMG) to differentiate structural from functional obstruction, and endoscopic evaluation are clinical diagnostic tools for voiding dysfunction (1, 11). PVR and bladder emptying efficiency are commonly used to assess treatment efficacy in voiding dysfunction, including BOO (12). Studies indicate that symptoms alone are unreliable for diagnosis of this dysfunction. Abnormal uroflowmetry and pressure-flow studies are essential to distinguish between impaired detrusor contractility and BOO. A maximum flow rate <15 mL/s and PVR >50 mL are reliable diagnostic markers for voiding dysfunction (9).

In voiding dysfunction, treatment aims to normalize voiding patterns and prevent complications. First-line therapies for functional voiding dysfunction include pelvic floor physiotherapy and biofeedback. Additional options (though lacking robust evidence) include psychological interventions, medications such as alpha-blockers, botulinum toxin injections, and GABA agonists (2). Intermittent catheterization (CIC) and electrical stimulation (invasive or noninvasive) are also employed (1).

Neuromodulatory electrical currents target lower urinary tract dysfunction by modulating neural control. Methods include pelvic floor electrical stimulation (vaginal, anal, or surface electrodes), interferential current (IFC), magnetic stimulation, percutaneous tibial nerve stimulation (PTNS), and sacral nerve stimulation (SNS) (13). These modalities enhance bladder capacity, relax pelvic muscles, and reduce bladder pressure (14). In voiding dysfunction, they increase bladder capacity due to the inhibition of pelvic reflexes. Electrical currents decrease sympathetic overactivity via pudendal nerve stimulation. So, they can reduce involuntary pelvic floor contractions. Because of essential role of pelvic floor muscles on sacral reflexes, stimulation of this region with IFC can impact on bladder function (15).

IFC employs two medium-frequency (1–10 kHz) out-of-phase sinusoidal currents. Their high frequency reduces skin impedance, generating a low-frequency interference current in deeper tissues (16). Session duration varies but typically lasts 20–30 minutes for non-acute conditions (17).

Despite clinical studies on IFC for pelvic disorders (e.g., urinary incontinence, constipation) (13, 18, 19), no research has examined its effects on female voiding dysfunction, though recent pediatric studies exist (14, 20). So, this study investigates IFC's effects on lower urinary tract symptoms (LUTS) and uroflowmetry in women with voiding dysfunction. Participants will be randomized into treatment and sham groups. Both will receive standard care (medication, urotherapy, and functional voiding exercises). Urotherapy includes education on urinary tract function, fluid intake, scheduled voiding (every 2–3 hours), and proper toileting. The treatment group will receive 10 sessions of real IFC, while the control group will undergo sham IFC. Post-treatment clinical assessment and clinical evaluations consist of uroflowmetry and PVR will use to investigate the treatment efficacy. If IFC proves effective for functional voiding dysfunction, it may emerge as a noninvasive therapeutic option.

Summary of the necessity of implementing the project

Regarding the use of interferential electric current as a current that modulates neural pathways in various pelvic floor disorders, including urinary incontinence, theoretical foundations support the use of this current based on increasing the activity of urethral sphincter afferents and causing relaxation in pelvic floor muscles in urinary incontinence (1, 2). Despite the existence of limited studies on the use of interferential current in urinary incontinence in the pediatric population, no study in this area was found in the female population with this disorder (1, 3). Given the impact on quality of life and the possibility of developing infection and chronic pain if

left untreated (4), the need to investigate the effect of interferential current in women with functional urinary retention as a non-invasive treatment option is clearly evident.

Summary of Methodology and Analysis

Study Design

Following ethical approval, a non-probability convenience sampling method will be employed. A urologist will evaluate patients with voiding dysfunction through:

- **Clinical history** (symptom assessment)
- **Neurological examination** (anal tone, voluntary anal control, genital/limb reflexes, sensory evaluation)
- **Urodynamic tests** (cystometry for detrusor pressure, uroflowmetry and pre-/post-void ultrasound to classify voiding dysfunction)

Eligible patients will complete a 3-day voiding diary (recording fluid intake, voiding frequency, and urinary patterns). They will then be referred to the physiotherapy clinic of rehabilitation faculty of Iran university of medical science with all of medical records. The urologist will remain blinded to group allocation. At the physiotherapy clinic, all participants will first receive a comprehensive explanation of the entire procedure and the purpose of the study. Then, individual informed consent forms will be obtained from each participant. Patients will be informed that there are two treatment groups, and except for the method of applying the interferential electrical current, all treatments in both groups are similar. Group assignment will be completely random, with an equal chance of being placed in either group. They will also be assured that if significant improvements are observed at the end of the study, the treatment received by the other group will be fully provided to them.

A completed 3-day urinary diary will be collected from each patient, and from this data, the frequency of bathroom visits within 24 hours will be extracted. Using a computerized random number list, patients will then be randomly assigned to either the treatment or control group in a 1:1 ratio, using blocks of four.

Next, a self-developed questionnaire collecting personal information such as age, height, and weight will be completed by all patients in both groups at the beginning of the first session. Afterward, all patients will receive the Persian versions of the female lower urinary tract symptom (FLUTS-ICIQ) and pelvic floor distress inventory-20 (PFDI-20) questionnaires to score the severity of their lower urinary tract symptoms and pelvic floor symptoms, respectively.

At the final treatment session, the Persian version of FLUTS-ICIQ and PFDI-20 questionnaires will be administered again to compare patients' scores on symptoms before and after treatment. Additionally, patients will complete the Persian version of the Patient Global Impression of Change (PGI-C) questionnaire to self-assess overall changes in their condition after treatment.

Finally, patients will be asked to complete the 3-day urinary diary once more. Two weeks after completing treatment, they will return for a visit with the urology specialist to undergo uroflowmetry testing, clinical examination, and evaluation of symptoms.

Moreover, a 3-month follow-up period is included in this study. After this period, uroflowmetry testing will be repeated once more, and clinical symptoms will be reassessed to evaluate the long-term effects of routine urotherapy alone versus routine urotherapy combined with IFC on clinical symptoms and uroflowmetry findings in patients with functional voiding dysfunction

Randomization and Blinding

- **Randomization**: Patients will be allocated with 1:1 ration into treatment (real IFC) or control (sham IFC) groups using computer-generated block randomization (block size = 4).
- **Blinding**:
    - **Patients**: Sham IFC will be applied for 1 minute (at perceptible but non-therapeutic intensity) to mimic active treatment. Real IFC will be applied with the strong tolerable intensity.
    - **Outcome assessor (urologist)**: Blinded to group assignment.
    - **Therapist**: Unblinded due to treatment nature.
- **Allocation concealment**: Sealed opaque envelopes (prepared by an independent person) will ensure concealment until intervention begins.

Statistical Analysis

Descriptive information regarding the demographic characteristics of individuals in both groups will be given as means (standard deviations) for quantitative variables and as frequencies (percentages) for qualitative variables. For comparing of each demographic variable between the two study groups, the independent t-test for normally distributed quantitative variables and the Mann-Whitney test for non-normally distributed quantitative variables will be applied. The chi-square test will be conducted to compare qualitative variables.

The normality of the data in the study groups will be tested using the Shapiro-Wilk test. To comprehensively examine the extracted data, the following procedures will be carried out, as elaborated below.

To compare the mean of each variable in both groups before the intervention, two weeks after the intervention, and after a three-month follow-up period, the two-way repeated measures ANOVA will be employed, assuming the data are normally distributed. In the case of non-normality, a nonparametric equivalent will be used.

For the comparison of the mean of the variables between both groups at every time point (before the intervention, at two weeks after the intervention, and after a three-month follow-up), the two independent sample t-test will be used if the data is normally distributed and the Mann-Whitney test if the data is distributed non-normally.

To determine differences in the mean of the variables of both groups over the study period, the one-way repeated measures ANOVA will be used. In the presence of a statistically significant difference in the variable mean over time, the post hoc test with Bonferroni and Tukey corrections will be utilized. The data will be analyzed using SPSS version 26 software. The significance level in this study is assumed to be 5%.

Ethical Considerations

Ethical Concerns and Issues in the Study:

1. Group allocation and differential treatments may lead to variations in treatment outcomes.

Solutions to Ethical Issues:

1. Prior to intervention, all patients will receive a full explanation of group allocation and treatments. All patients will be assured that if any clinically significant improvement is demonstrated in either group, the effective treatment will be provided to the other group with the same duration and quality after study completion.
2. Participation is entirely voluntary. Written informed consent will be obtained from all patients before treatment initiation.
3. Principles of confidentiality and data protection will be strictly maintained.
4. All participants retain the right to withdraw from the study at any stage without consequences.

Does the study have ethical approval?

Yes (code: IR.IUMS.REC.1403.547)

Research Questions & Hypotheses

(All hypotheses are stated as null hypotheses.)

1. PVR:
   - H_0: There is no significant difference in mean PVR before and after treatment in the real IFC group.
   - H_0: There is no significant difference in mean PVR after treatment between the real IFC and sham IFC groups.
2. Qmax:
   - H_0: There is no significant difference in mean Qmax before and after treatment in the real IFC group.
   - H_0: There is no significant difference in mean Qmax after treatment between the real IFC and sham IFC groups.
3. 24-Hour Urinary Frequency:
   - H_0: There is no significant difference in mean 24-hour urinary frequency before and after treatment in the real IFC group.
   - H_0: There is no significant difference in mean 24-hour urinary frequency after treatment between the real IFC and sham IFC groups.
4. LUTS Severity (ICIQ-FLUTS):
   - H_0: There is no significant difference in mean ICIQ-FLUTS scores before and after treatment in the real IFC group.
   - H_0: There is no significant difference in mean ICIQ-FLUTS scores after treatment between the real IFC and sham IFC groups.
5. Pelvic floor symptom severity (PFDI-20):
   - H_0: There is no significant difference in mean PFDI-20 scores before and after treatment in the real IFC group.
   - H_0: There is no significant difference in mean PFDI-20 scores after treatment between the real IFC and sham IFC groups.
6. Patient-Reported Improvement (PGIC):
   - H_0: There is no significant difference in mean PGIC scores after treatment between the real IFC and sham IFC groups.

Definitions of Key Terms

1. Voiding Dysfunction

- Conceptual Definition: A set of symptoms experienced during or after urination, including: weak urinary stream, splitting/spraying of urine, intermittent flow, difficulty initiating voiding, straining to void, sensation of incomplete emptying, prolonged terminal dribbling, post-void dribbling, need for immediate re-voiding and urination only in specific positions (8, 21).
- Operational Definition: In this study, patients diagnosed with voiding dysfunction by a referring urologist will be enrolled.

2. Obstructive Voiding Dysfunction

- **Conceptual Definition**: Reduced urinary flow rate (Qmax <15 mL/s) and elevated detrusor pressure (>20 cm H_2O) during voiding in the absence of infection or overt pathology. It is diagnosed via pressure-flow study (PFS) and uroflowmetry and may be associated with increased post-void residual (PVR) (6, 8). The urodynamic characteristics in this dysfunction are as following:
    - Filling phase: Normal detrusor stability (no involuntary contractions).
    - Voiding phase: Electromyographic (EMG) activity in pelvic floor muscles; variable PVR (22).
    - Bladder Outlet Obstruction Index (BOOI): A value >18 supports diagnosis (12).
- **Operational Definition**: In this study, patients with functional obstructive voiding dysfunction will be diagnosed by a urologist. Diagnostic criteria: BOOI, Qmax, voiding time (urodynamic testing) and PVR (ultrasound).

3. Urinary Incontinence

- **Conceptual Definition (ICS Standards)**: involuntary urine leakage which affects social life and quality of life of patient. It includes 3 categories:
    - Stress urinary incontinence (SUI): Leakage due to increased intra-abdominal pressure (e.g., coughing, sneezing) (23, 24)
    - Urge urinary incontinence (UUI): Leakage preceded by urgency.
    - Mixed urinary incontinence (MUI): Combined SUI and UUI symptoms (23).
- **Operational Definition**:
  Patients must answer "yes" to at least one screening question:

1. *"In the past month, have you experienced urine leakage after specific physical activity (e.g., lifting, laughing, coughing)?"*
2. *"In the past month, have you had sudden urges to urinate that were difficult to control?"* (25).

4. Urotherapy

- **Conceptual Definition**:
  Non-surgical and non-pharmacological interventions to normalize urinary/defecation patterns and prevent functional disorders through repetitive training (26).
- **Operational Definition**:
  Standard urotherapy in this study includes:
  - Education on urinary tract function.
  - Fluid intake management.
  - Scheduled voiding (every 2–3 hours).
  - Proper toileting posture (spinal alignment, relaxed pelvic floor).
  - Instruction on intermittent catheterization (if needed).
  - Pelvic floor muscle training: 10-second contractions followed by 30-second relaxation.
  - High-fiber diet and regular bowel habits (1, 3).

5. Interferential Current (IFC)

- **Conceptual Definition**:
  A low-frequency (1–100 Hz), amplitude-modulated current generated by interference of two medium-frequency (1–10 kHz) currents. It penetrates deep tissues by overcoming skin impedance (16, 18). It is Recommended to use this current for 20–30 minutes per session for non-acute conditions (19).
- **Operational Definition**:
  - Device: Standard electrotherapy unit (Novin, Iran).
  - Parameters:
    - Carrier frequency: 4,000 Hz
    - Beat frequency: 80–160 Hz
    - Duration: 20 minutes (pelvic region).

- Electrode placement: Cross-shaped over pubic symphysis and ischial tuberosities.
- Intervention protocol:
    - Real IFC group: Current intensity adjusted as high as possible according to patient tolerance.
    - Sham IFC group: Current applied for 1 minute in the first sensation of patient, then discontinued (27).

Variables

Severity of Lower Urinary Tract Symptoms (LUTS)

- **Conceptual Definition**:
  Subjective symptoms perceived by the patient, caregiver, or family, prompting medical consultation. These qualitative symptoms are typically identified during patient history-taking (23).
- **Operational Definition**:
  Assessed using the Persian version of the ICIQ-FLUTS questionnaire, which categorizes symptoms into:
    - Storage-phase symptoms
    - Voiding-phase symptoms (dependent variable)
    - Urinary incontinence symptoms (confounding variable).

Post-Void Residual (PVR) Volume

- **Conceptual Definition**:
  The volume of urine remaining in the bladder after complete voiding without catheterization (10). PVR correlates with bladder capacity; higher pre-void volumes yield higher PVR (15). It should be performed ≤60 seconds post-void via ultrasound to minimize refilling artifacts (bladder refills at 1–14 mL/sec) (10). PVR >50 mL suggests voiding dysfunction (13).
- **Operational Definition**:
  it will be measured by pre- and post-void ultrasound performed by a urologist (13).

Maximum Urinary Flow Rate (Qmax)

- **Conceptual Definition**:
  The peak flow rate (mL/sec) during voiding, denoted as Qmax (10). Qmax <15 mL/sec in women indicates voiding dysfunction (13).

- **Operational Definition**:
  Recorded during **uroflowmetry** by a urologist.

Voiding Time

- **Conceptual Definition**:
  Total duration of urination, including interruptions. It matches flow time if voiding is continuous (10).
- **Operational Definition**:
  Extracted from **uroflowmetry** reports.

Urinary Flow Pattern

- **Conceptual Definition**:
  The shape of the flow curve on uroflowmetry:
    - **Normal**: Bell-shaped, continuous.
    - **Abnormal**:
        - **Obstructive**: Flattened/prolonged.
        - **Detrusor-sphincter dyssynergia (DSD)**: Intermittent/spiky (11, 23).
- **Operational Definition**:
  Obtained from **uroflowmetry** (pre- and post-treatment) by a urologist.

Patient Global Impression of Change (PGIC)

- **Conceptual Definition**:
  A global assessment tool for any patient-reported changes (improvement or deterioration) post-treatment (28).
- **Operational Definition**:
  Evaluated using the PGI-C questionnaire post-intervention.

Literature Review

1. Kajbafzadeh et al. (2015) (20)

Study Design:
Interventional study on 36 children (5-13 years) with underactive bladder (UAB), divided into two groups:

- **Group 1:** Standard urotherapy alone (n=18)
- **Group 2:** Standard urotherapy + interferential current (IFC) (n=18)

Inclusion Criteria:

- Urodynamically confirmed UAB
- Poor response to pharmacotherapy
- Intermittent urinary flow pattern, low flow rate (<15 mL/s), prolonged voiding time, voiding frequency <3/day, straining, PVR >20 mL

Intervention:

- **Standard urotherapy:** Education on urinary tract function, fluid intake, scheduled voiding (every 2–3 hours), proper toileting posture.
- **IFC:** 20-minute sessions (carrier frequency: 4,000 Hz) using cross-shaped electrode placement (pubic symphysis + ischial tuberosities).

Outcomes:

- **IFC group showed:**
  - Significant reduction in PVR ($p<0.05$)
  - Increased voiding frequency ($p<0.05$)
  - Improved Qmax and reduced voiding time ($p<0.05$)
  - 77% normalized urinary flow pattern vs. 33% in urotherapy-only group.

Conclusion:

IFC enhances bladder emptying in pediatric UAB by modulating neuromuscular function.

2. Sharifi-Rad et al. (2019) (14)

Study Design:

Interventional study on 23 neurologically intact children with primary bladder neck dysfunction (PBND).

Inclusion Criteria:

- Symptoms >6 months, poor drug response
- Abnormal uroflowmetry (flat curve, delayed EMG silence >6 sec, low Qmax)

Intervention:

- **15 IFC sessions** (4,000 Hz, 20 min/session) + standard urotherapy.

Results:

- **Significant improvements in:**
    - Qmax (*p<0.01*)
    - PVR (*p<0.01*)
    - EMG silence latency (*p<0.01*)

Conclusion:
IFC mitigates functional bladder outlet obstruction in PBND.

3. Ladi Seyedian et al. (2020) (29)

Study Design:
Randomized trial on 34 children with non-neurogenic bowel-bladder dysfunction (BBD).

Groups:

- **IFC + pelvic floor exercises (PFE)** (n=17)
- **PFE alone** (n=17)

Outcomes:

- **IFC+PFE group:**
    - 64.7% achieved "complete response" (vs. 29.4% in PFE group) (*p<0.05*)
    - Reduced urinary incontinence episodes (*p<0.05*)

Limitation:
No significant uroflowmetry differences post-treatment (23).

4. Vitton et al. (2022) (18)

Study Design:
Double-blind RCT on adults with chronic constipation.

Intervention:

- **IFC (4,000 Hz, 80–160 Hz)** vs. sham (8 weeks, 1 hr/day).

Results:

- Improved PAC-SYM (*$p<0.01$*) and PAC-QOL (*$p<0.05$*) scores.
- **No difference** in bowel motility or manometry.

Conclusion:
IFC alleviates symptoms but lacks mechanistic impact.

5.Moore et al. (2019) (30)

Study Design:
Single-blind RCT on 33 women with functional constipation.

Intervention:

- IFC (abdominal-posterior electrode placement) vs. sham (lateral placement).

Results:

- 53% of IFC group achieved ≥3 spontaneous bowel movements/week (vs. 12.5% sham) (*$p<0.05$*).
- Reduced laxative use (*$p<0.05$*) but no PAC-SYM improvement.

Critique:
Methodological limitations noted in systematic reviews.

Critical Synthesis

- **Pediatric Studies:** Strong evidence for IFC in voiding dysfunction, with improvements in PVR, Qmax, and symptoms.
- **Adult Studies:** IFC improves subjective symptoms (constipation) but lacks objective urodynamic/physiological changes.
- **Gaps:** Limited adult data on IFC for urinary voiding disorders.

Is this project a thesis?

It is not a thesis.

1. Participant Recruitment and Screening

- **Sampling Method:** Non-probability convenience sampling after ethics approval.
- **Screening Process:**
  - Conducted by urologists at outpatient clinic.
  - Comprehensive evaluation including:

        - Medical history and symptom assessment
        - Neurological exam (anal tone, voluntary anal control, genital/lower limb reflexes)
        - Urodynamic tests (cystometry for detrusor pressure, uroflowmetry)
        - Pre-/post-void bladder ultrasound

- **Inclusion Criteria:** Patients meeting diagnostic criteria for voiding dysfunction.
- **Baseline Data Collection:**
    - 3-day voiding diary (fluid intake, voiding frequency)
    - Standard medical record documentation (urodynamics, ultrasound reports).

2. Randomization and Blinding

- **Randomization:**
    - Computer-generated block randomization (1:1 ratio, block size=4).
    - Stratified by age and symptom severity.
- **Blinding:**
    - **Patients:** Sham-controlled (details below).
    - **Outcome Assessor (Urologist):** Blinded to group allocation.
    - **Therapist:** Unblinded due to intervention nature.

Presenters and colleagues

Project Implementation Timeline

#	Phase	Duration	Monthly Progress Tracking (1-10)
1	Proposal Writing	1 month	10 9 8 7 6 5 4 3 2 **1**
2	Proposal Approval & Ethical Code Acquisition	1 month	10 9 8 7 6 5 4 3 **2** 1

#	Phase	Duration	Monthly Progress Tracking (1-10)
3	Implementation & Data Collection	6 months	10 9 **8 7 6 5 4 3** 2 1
4	Data Analysis	1 month	10 **9** 8 7 6 5 4 3 2 1
5	Results Reporting & Article Publication	1 month	**10** 9 8 7 6 5 4 3 2 1

3. Interventions

All Participants Receive:

- **Standard Urotherapy (10 sessions, 20 mins/session, 2x/week):**
  - Education on urinary tract function
  - Fluid management (1.5–2 L/day)
  - Scheduled voiding (every 2–3 hours)
  - Proper toileting posture (spinal alignment, foot support)
  - Pelvic floor muscle training (10-sec contraction + 30-sec relaxation).

Group-Specific Protocols:

Component	Active IFC Group	Sham IFC Group
**Device**	Novin 2-channel (Iran)	Identical device
**Parameters**	4,000 Hz carrier, 80Â–160 Hz beat	Same setup
**Electrode Placement**	Cross-shaped (pubic symphysis + ischial tuberosities)	Identical placement
**Intensity**	Strong but comfortable	Initial sensation (1 min), then OFF

Component	Active IFC Group	Sham IFC Group
**Session Duration**	30 mins IFC + 20 mins urotherapy	1 min IFC + 29 mins sham

4. Outcome Measures

Primary Endpoints:

1. **Uroflowmetry:** Qmax, voiding time, PVR (ultrasound).
2. **ICIQ-FLUTS:** Symptom severity.

Secondary Endpoints:

- PFDI-20 (pelvic floor distress).
- PGIC (patient-reported improvement).
- 3-day voiding diary (24-hour frequency).

Assessment Timeline:

- **Baseline:** Pre-treatment.
- **Post-Treatment:** After 10 sessions (5 weeks).
- **Follow-Up:** 3 months post-treatment.

6. Ethical Considerations

- **Informed Consent:** Obtained prior to randomization.
- **Sham Control:** Ethical approval for minimal IFC exposure (1 min).
- **Post-Trial Access:** Active treatment offered to sham group if significant benefits observed.

Key Methodological Strengths

1. **Robust Blinding:** Sham protocol minimizes placebo effects.
2. **Standardized Protocols:** Identical urotherapy for both groups ensures comparability.
3. **Objective + Subjective Measures:** Combines urodynamics (Qmax, PVR) with validated questionnaires (ICIQ-FLUTS).
4. **Longitudinal Design:** 3-month follow-up assesses sustainability.

Limitations:

This study may encounter patient attrition due to the follow-up period. In order to reduce this issue, an effort has been made to keep the follow-up period as short as possible and to clearly explain to patients the importance of returning to a urologist after the follow-up period.

Protocol Deviations Management

- **Non-Adherence:** Participants missing >2 sessions excluded from per-protocol analysis.
- **Adverse Events:** Documented and reported to ethics board (e.g., skin irritation from electrodes).

This protocol aligns with CONSORT guidelines for non-pharmacological trials. Let me know if you need additional operational details!

Specifications of Data Collection Tools and Collection Methods

1) Researcher-Developed Questionnaire for Collecting Demographic Information

A questionnaire designed by the researchers of this study to collect patients' demographic characteristics including age, height, weight, and body mass index (BMI).

2) Uroflowmetry Test

Urodynamic studies are considered the gold standard for diagnosing urinary voiding disorders. This test provides a functional assessment of the lower urinary tract, beginning with simple non-catheter uroflowmetry followed by measurement of post-void residual urine volume using full and empty bladder ultrasound (10). Uroflowmetry is a non-invasive test that evaluates urinary voiding function before treatment and during follow-ups. In this test, the patient urinates into a special device that measures urine flow rate. At the end of the test, a urine flow curve is plotted and quantitative parameters are reported (11, 31).

3) 3-Day Voiding Diary (32)

A form where the patient records the number of urinations, volume of urine voided each time, incontinence episodes, pad usage (in cases of incontinence), catheter use (in cases of urinary retention), as well as the amount and type of fluids consumed over three days. For retention disorders, using a 3-day or longer voiding diary is recommended (9, 33).

4) PFDI-20 Questionnaire (34)

This questionnaire, recommended by the International Continence Society, contains 20 questions across three different domains assessing the severity of pelvic floor disorders. The total score is 300, with each domain scored out of 100, where higher scores indicate more severe symptoms.

5) Persian Version of ICIQ-FLUTS Questionnaire (35)

A validated questionnaire for assessing lower urinary tract symptoms and their impact on quality of life, containing 12 questions across three domains: filling phase symptoms, voiding phase symptoms, and incontinence symptoms.

6) GRCS Questionnaire

A questionnaire for evaluating patient changes after treatment, where patients rate their changes on a 7-point scale from "much worse" to "much better" (33.

Sample Size Calculation Method

Based on the study by Kajbafzadeh et al. and considering a 95% confidence level and 80% test power, the sample size was calculated as 14 participants per group (20).

Study Limitations and Mitigation Strategies

The main limitation of the study is potential sample attrition due to the follow-up period. To minimize this issue, a short follow-up duration was selected, and the importance of follow-up visits was thoroughly explained to patients.

Does the research involve specialized tests or specific techniques?

Yes

Knowledge Translation

Target Audience:

- Healthcare providers (physicians, nurses, midwives, etc.)

If the target audience is policymakers/administrators:

[No explanation provided]

If the target audience is healthcare providers:

If interferential current therapy proves effective in significantly improving clinical symptoms and urodynamic findings in women with voiding dysfunction, pelvic floor disorder specialists (physiotherapists, urologists, pelvic medicine fellows,

gynecologists) could incorporate this treatment into their practice for patients with urinary disorders.

If the target audience is the general public/patients:

[No explanation provided]

If the target audience is companies/industries:

[No explanation provided]

If the target audience is other organizations:

[No explanation provided]

Knowledge Dissemination Methods:

- Presentation at international conferences/seminars
- Presentation at domestic conferences/seminars
- Sending summary/full report or resulting articles to potential users
- Publication in international scientific-research journals
- Publication in domestic scientific-research journals
- Posting full report/summary on website for potential users

Explanation of Implementation Methods:

All therapeutic groups in pelvic floor disorders including physiotherapists, urologists, and gynecologists can benefit from the study results for treating voiding dysfunction. Physiotherapists can use the mentioned methods upon physician referral and add effective treatment protocols. The principal investigator, as a pelvic floor specialist physiotherapist, can utilize superficial electrical stimulation in specified areas to improve voiding dysfunction after publishing results and confirming treatment efficacy.

Primary Stakeholder (Individual/Organization):

Patients with voiding dysfunction

- Importance score (1-5): 5
- Power score (1-5): 2

Secondary Stakeholder:

Physiotherapy department, Rehabilitation Faculty, Iran University of Medical Sciences

- Importance score: 3
- Power score: 2

Tertiary Stakeholder:

Pelvic floor disorder therapists and physiotherapists

- Importance score: 5
- Power score: 1

Quaternary Stakeholder:

Urologists, gynecologists, and pelvic medicine fellows

- Importance score: 4
- Power score: 3

Design variables

**Variable Name**	**Variable Role**	**Variable Type**	**Scientific Definition**	**Measurement Method**	**Scale**
Post-void residual volume (PVR)	Dependent	Quantitative/Continuous	Refers to urine remaining in the bladder after voiding. Determined using catheterization after complete voiding (5,11). PVR >100 mL suggests urinary retention (20).	Ultrasound	mL

Variable Name	Variable Role	Variable Type	Scientific Definition	Measurement Method	Scale
Maximum urinary flow rate (Qmax)	Dependent	Quantitative/Continuous	The peak urinary flow rate, denoted as Qmax in studies, represents the highest measured urine flow velocity reported in mL/sec (10).<br>Reduced Qmax indicates voiding dysfunction. Values <15 mL/sec in women suggest voiding dysfunction (13).	Uroflowmetry	mL/sec
Toilet use frequency	Dependent	Quantitative/Discrete	-	3-day voiding diary	-
Lower urinary tract symptom severity	Dependent	Quantitative/Continuous	Subjective symptoms perceived by patients/caregivers that prompt treatment-seeking, typically obtained through history-taking (1).	Persian ICIQ-FLUTS	-
Patient-reported treatment changes	Dependent	Quantitative/Discrete	Global assessment of disease status using standardized indices that report overall symptom	Persian PGI-C	-

Variable Name	Variable Role	Variable Type	Scientific Definition	Measurement Method	Scale
			changes post-treatment (33).		
Urinary incontinence symptom severity	Background	Quantitative/Continuous	Subjective symptoms prompting treatment-seeking, typically qualitative data from patient history (27).	Persian ICIQ-FLUTS LF	-
Age	Background	Quantitative/Discrete	Chronological age calculated from birth date in years/months/days	Questionnaire	years
Weight	Background	Quantitative/Continuous	The mass of a person's body	Standard scale	kg
Height	Background	Quantitative/Continuous	Vertical distance from foot to head in standing position	Measuring tape	cm

Travel Expenses

No data recorded for travel expenses.

Other Expenses

**Cost Item**	**Amount (IRR)**	**Total**
Printing and duplication of questionnaires (based on sample size and number of repetitions)	30,000,000	30,000,000
**Grand Total**		**30,000,000**

Cost of tests and specialized services

**Test Subject**	**Test Type**	**Cost per Test (IRR)**	**Test Location**	**Number of Tests**	**Total (IRR)**
Uroflowmetry	Device and materials	4,500,000	Iran University of Medical Sciences hospitals	30	135,000,000
Electrical stimulation	Device	400,000	Rehabilitation Sciences Faculty Physiotherapy Clinic	300	120,000,000
Ultrasound	Device and materials	1,500,000	Ultrasound Clinic	30	45,000,000
**Grand Total**					**300,000,000**

Cost of purchased equipment and materials

Equipment Name	Consumable/Non-Consumable	Quantity	Unit Price (IRR)	Manufacturer	Unit	Total (IRR)
Electrotherapy pads	Consumable (Usage)	60	700,000	Nahal	Number	42,000,000
**Grand Total**						**42,000,000**

Financing

Funding Type	Funding Institution/Organization/Center/Faculty	Payment Method	Amount Funded by Target Center (IRR)	Externally Funded Amount (IRR)	Total (IRR)
Common within the university	Iran University of Medical Sciences	-	0	0	0
**Grand Total**					**0**

Researcher's Commitment:
I hereby confirm that I have not initiated this study prior to obtaining approval from the university ethics committee. If informed consent is required for my study, I commit to submitting 10% of completed consent forms along with contact information and names of study participants when submitting the first report.
Yes

Consent Form

1. I understand the study objectives are:
   To determine and compare the effects of interferential current therapy and standard urotherapy on clinical symptoms and urodynamic findings in women with voiding dysfunction.

2. I understand my participation is completely voluntary and I am under no obligation to participate. I have been assured that refusal to participate will not affect my access to standard diagnostic and therapeutic care, nor my relationship with the treating physician or medical center.

3. I understand I may withdraw from the study at any time after notifying the researcher, without compromising my access to standard medical care.

4. My involvement in the study will include:
   Participation in treatment sessions including standard urotherapy exercises and real/sham electrical stimulation. Both groups will receive 10 treatment sessions (2 sessions/week). Real/sham electrical stimulation will be administered for 20 minutes per session, with the remainder dedicated to urotherapy and therapeutic exercises.

5. Potential benefits of participation:

- Improvement in urinary symptoms and quality of life
- Contribution to advancing treatment knowledge for urinary disorders

6. Potential risks/side effects:

- The study uses approved electrotherapy protocols with minimal risks (possible minor skin redness at electrode sites)
- If symptoms worsen, immediate urology referral will be provided

7. Alternative standard treatment options:
   Pharmacological management of urinary disorders with its specific benefits and side effects

8. I understand all my personal information will remain confidential, with only aggregated results being published without identifying details.

9. I acknowledge the ethics committee may access my information to monitor compliance with my rights.
10. I understand I will not bear any costs for:

- Physiotherapy interventions (interferential current therapy)

11. Contact information for study inquiries:

12. **Compensation for Adverse Effects:**
I understand that if any physical or psychological complications arise during or after the study due to my participation, the **principal investigator** will be responsible for covering:

- Medical treatment for such complications
- Any related compensation

13. **Complaint/Grievance Redressal:**
I am aware that if I have any objections or concerns regarding the study procedures or investigators, I may contact the **Ethics Committee of Iran University of Medical Sciences** at:
Address:
Central Building, 5th Floor (Vice-Chancellor for Research & Technology)
Iran University of Medical Sciences, Hemmat West Highway
Between Sheikh Fazlollah and Chamran Intersections, Tehran
Tel: (+98) 21-86702530
Complaints may be submitted **orally or in writing**.

14. **Documentation:**
This informed consent form has been prepared in **two identical copies**. After signing:

- One copy will remain with **me (the participant)**
- The other copy will be retained by the **principal investigator**

The sum total of the costs

**Cost Item**	**Total (IRR)**
Total Personnel Costs	128,500,000

Cost Item	Total (IRR)
Specialized Tests and Services Costs	300,000,000
Equipment and Materials Purchases	42,000,000
Travel Expenses	0
Other Expenses	30,000,000
Funding from Other Organizations	0
**Total Costs**	**500,500,000**
Budget Provided by Internal Partner (Second Center)	0
Deduction of Allocated Funding from Other Organizations	0
**Required Budget (Total Without External Funding)**	**500,500,000**

1. Abdel Raheem A, Madersbacher H. Voiding dysfunction in women: How to manage it correctly. Arab journal of urology. 2013;11(4):319-30.
2. Artibani W, Cerruto MA. Dysfunctional voiding. Current opinion in urology. 2014;24(4):330-5.
3. Yang TH, Chuang FC, Kuo HC. Urodynamic characteristics of detrusor underactivity in women with voiding dysfunction. PloS one. 2018;13(6):e0198764.
4. Chapple CR, Osman NI, Birder L, Dmochowski R, Drake MJ, van Koeveringe G, et al. Terminology report from the international continence society (ICS) working group on underactive bladder (UAB). 2018;37(8):2928-31.
5. Abrams P, Cardozo L, Fall M, Griffiths D, Rosier P, Ulmsten U, et al. The standardization of terminology of lower urinary tract function: report from the standardization sub-committee of International Continence Society. Textbook of Female Urology and Urogynecology: CRC Press; 2010. p. 1098-108.
6. Haylen BT, De Ridder D, Freeman RM, Swift SE, Berghmans B, Lee J, et al. An International Urogynecological Association (IUGA)/International Continence Society (ICS) joint report on the terminology for female pelvic floor dysfunction. 2010;29(1):4-20.

7. Pfeiffer RF. CHAPTER 29 - BLADDER AND SEXUAL FUNCTION AND DYSFUNCTION. In: Schapira AHV, Byrne E, DiMauro S, Frackowiak RSJ, Johnson RT, Mizuno Y, et al., editors. Neurology and Clinical Neuroscience. Philadelphia: Mosby; 2007. p. 362-71.
8. Santis-Moya F, Calvo CI, Rojas T, Dell'Oro A, Baquedano P, Saavedra A. Urodynamic and clinical features in women with overactive bladder: When to suspect concomitant voiding dysfunction? Neurourology and urodynamics. 2021;40(6):1509-14.
9. Lemack GE. Urodynamic assessment of bladder-outlet obstruction in women. Nature clinical practice Urology. 2006;3(1):38-44.
10. Uren AD, Drake MJ. Definition and symptoms of underactive bladder. Investigative and clinical urology. 2017;58(Suppl 2):S61-s7.
11. Abrams P, Andersson K-E, Birder L, Brubaker L, Cardozo L, Chapple C, et al. Fourth International Consultation on Incontinence Recommendations of the International Scientific Committee: Evaluation and treatment of urinary incontinence, pelvic organ prolapse, and fecal incontinence. 2010;29(1):213-40.
12. Yono M, Ito K, Oyama M, Tanaka T, Irie S, Matsukawa Y, et al. Variability of post-void residual urine volume and bladder voiding efficiency in patients with underactive bladder. Lower urinary tract symptoms. 2021;13(1):51-5.
13. Yamanishi T, Kaga K, Fuse M, Shibata C, Uchiyama TJLLUTS. Neuromodulation for the treatment of lower urinary tract symptoms. 2015;7(3):121-32.
14. Sharifi-Rad L, Seyedian S-SL, Fatemi-Behbahani S-M, Lotfi B, Kajbafzadeh A-MJJoPU. Impact of transcutaneous interferential electrical stimulation for management of primary bladder neck dysfunction in children. 2020;16(1):36. e1-. e6.
15. Sharifi-Rad L, Ladi-Seyedian S-S, Kajbafzadeh A-MJUJ. Interferential Electrical Stimulation Efficacy in the Management of Lower Urinary Tract Dysfunction in Children: A Review of the Literature. 2021;18(5).
16. Rampazo É P, Liebano RE. Analgesic Effects of Interferential Current Therapy: A Narrative Review. Medicina (Kaunas, Lithuania). 2022;58(1).
17. Watson T. *Interferential Therapy (IFT)*. 2015.
18. Vitton V, Mion F, Leroi AM, Brochard C, Coffin B, Zerbib F, et al. Interferential therapy for chronic constipation in adults: The CON-COUR randomizedcontrolled trial. United European gastroenterology journal. 2023;11(4):337-49.
19. Southwell BR. Medical devices to deliver transcutaneous electrical stimulation using interferential current to treat constipation. Expert review of medical devices. 2013;10(6):701-4.
20. Kajbafzadeh AM, Sharifi-Rad L, Ladi-Seyedian SS, Mozafarpour SJBi. Transcutaneous interferential electrical stimulation for the management of non-neuropathic underactive bladder in children: a randomised clinical trial. 2016;117(5):793-800.
21. Bosch JRJBi. Electrical neuromodulatory therapy in female voiding dysfunction. 2006;98:43-8.
22. Schulman SL, Quinn CK, Plachter N, Kodman-Jones CJP. Comprehensive management of dysfunctional voiding. 1999;103(3):e31-e.
23. Abrams P, Cardozo L, Fall M, Griffiths D, Rosier P, Ulmsten U, et al. The standardisation of terminology in lower urinary tract function: report from the standardisation sub-committee of the International Continence Society. 2003;61(1):37-49.
24. Jin X, Tang H, Chen G. CT Three-Dimensional Visualization Model in Diagnosis and Treatment of Stress Urinary Incontinence: A Retrospective Study. Urology. 2023;172:84-8.
25. Rohr G, Christensen K, Ulstrup K, Kragstrup J. Reproducibility and validity of simple questions to identify urinary incontinence in elderly women. Acta obstetricia et gynecologica Scandinavica. 2004;83(10):969-72.

26. Nieuwhof-Leppink AJ, Hussong J, Chase J, Larsson J, Renson C, Hoebeke P, et al. Definitions, indications and practice of urotherapy in children and adolescents: - A standardization document of the International Children's Continence Society (ICCS). Journal of pediatric urology. 2021;17(2):172-81.
27. Rakel B, Cooper N, Adams HJ, Messer BR, Frey Law LA, Dannen DR, et al. A new transient sham TENS device allows for investigator blinding while delivering a true placebo treatment. The journal of pain. 2010;11(3):230-8.
28. Barber MD. Questionnaires for women with pelvic floor disorders. International urogynecology journal and pelvic floor dysfunction. 2007;18(4):461-5.
29. Ladi-Seyedian SS, Sharifi-Rad L, Kajbafzadeh AM. Management of Bladder Bowel Dysfunction in Children by Pelvic Floor Interferential Electrical Stimulation and Muscle Exercises: A Randomized Clinical Trial. Urology. 2020;144:182-7.
30. Moore JS, Gibson PR, Burgell RE. Randomised clinical trial: transabdominal interferential electrical stimulation vs sham stimulation in women with functional constipation. Alimentary pharmacology & therapeutics. 2020;51(8):760-9.
31. Kim M, Jeong CW, Oh SJ. Diagnostic value of urodynamic bladder outlet obstruction to select patients for transurethral surgery of the prostate: Systematic review and meta-analysis. PloS one. 2017;12(2):e0172590.
32. Tayebi S, Salehi-Pourmehr H, Hajebrahimi S, Hashim H. Translation and validation of the Persian ICIQ bladder diary. International urogynecology journal. 2021;32(12):3287-91.
33. Lewis AL, Young GJ, Abrams P, Blair PS, Chapple C, Glazener CMA, et al. Clinical and Patient-reported Outcome Measures in Men Referred for Consideration of Surgery to Treat Lower Urinary Tract Symptoms: Baseline Results and Diagnostic Findings of the Urodynamics for Prostate Surgery Trial; Randomised Evaluation of Assessment Methods (UPSTREAM). European urology focus. 2019;5(3):340-50.
34. Hakimi S, Hajebrahimi S, Bastani P, Aminian E, Ghana S, Mohammadi MJBo. 208: translation and validation of the pelvic floor distress inventory short form (PFDI-20), Iranian version. 2017;7(Suppl 1):bmjopen-2016-015415.208.
35. Pourmomeny A, Alebouye-Langeroudi S, Zargham M. Reliability and Validity of the Persian Language Version of the Female Lower Urinary Tract Symptoms' Long form Questionnaire. Iranian journal of nursing and midwifery research. 2018;23(6):421-5.
