# Supplementary material for: Effects of interferential stimulation on clinical symptom and urodynamic findings in women with voiding dysfunction: A protocol of randomized clinical trial
Source: PLoS One. 2025 Oct 27;20(10):e0330610. doi: 10.1371/journal.pone.0330610 (PMC12558555; doi:10.1371/journal.pone.0330610)
Supplement: S2 File — (PDF) [file pone.0330610.s003.pdf]

عنوان طرح گرنت : بررسی تاثیر جریان اینترفرنشیال بر علائم بالینی و یافته های یورودینامیک در زنان مبتلا به اختلال تخلیه ادرار: یک کارآزمایی بالینی

Title : Investigating the effects of interferential stimulation on clinical symptom and urodynamic findings in women with voiding dysfunction: A randomized clinical trial

کد رهگیری: ۲۸۲۵۱

پژوهشگر : سیده سعیده بابازاده

تخصص: فیزیوتراپی

تاریخ ثبت اولیه: ۰۹/۱۱/۱۴۰۲ ۱۴:۲۳:۰۹

تاریخ ارسال: ۱۶/۱۲/۱۴۰۲ ۲۲:۲۶:۵۳

تاریخ این ویراست: ۰۵/۰۶/۱۴۰۳ ۰۹:۵۴:۲۴

مرکز/دانشکده بررسی کننده اصلی: دانشگاه علوم پزشکی ایران

مرکز هدف دوم: دانشکده علوم توانبخشی

مشخصات کلی طرح و چکیده

نوع گرنت

گرنت اولین پروپوزال ارسالی

حیطه

سایر

اولویت

سایر

آیا طرح مطالعه حیوانی است؟

خیر

عنوان فارسی

بررسی تاثیر جریان اینترفرنشیال بر علائم بالینی و یافته های یورودینامیک در زنان مبتلا به اختلال تخلیه ادرار: یک کارآزمایی بالینی

آیا طرح کارآزمایی بالینی میباشد؟

بله

پاسخگویی اجتماعی دانشگاه

خیر

عنوان انگلیسی

Investigating the effects of interferential stimulation on clinical symptom and urodynamic findings in women with voiding dysfunction: A randomized clinical trial

---

کلیدواژه های انتخابی

لاین تحقیقاتی

فیزیوتراپی کف لگن

کلید واژه

اختلال تخلیه ادرار، اختلال تخلیه ادرار عملکردی، جریان الکتریکی اینترفرنشیال، جریان اینترفرنشیال، یورودینامیک، یوروفلومتری، زنان

نوع طرح

Practical research - عملی

مقدمه-بیان مساله

مقدمه

اختلال در تخلیه کامل ادرار یک مشکل کلینیکی شایع در زنان می باشد که مرتبط با تخلیه نامناسب مثانه است(۱). بعلت روش کار و تعاریف متفاوت استفاده شده در مطالعات مختلف، تخمین درستی از شیوع این اختلال وجود ندارد، اما براساس یافته های یورودینامیک تا ۲ درصد از بزرگسالان درگیر این اختلال هستند(۲). شیوع اختلالات تخلیه ادرار در زنان بسته به شاخص های تشخیصی مختلف، بین ۶/۸ تا ۶۱/۷ درصد تخمین زده شده است که با افزایش سن این میزان نیز بیشتر میشود(۳). این اختلال شامل علائمی است که در فاز تخلیه یا پس از تخلیه تجربه میشوند. از میان همه علائم، در درجه اول احساس تخلیه ناکامل و بعد از آن جریان ضعیف ادرار دو علامت شایع مورد گزارش توسط بیماران هستند. این اختلال گاهی میتواند همراه با علائم فاز ذخیره ادرار باشد که در اینصورت پاتولوژی هر کدام میتواند مستقل یا مرتبط با هم در فرد رخ داده باشد (۱, ۲, ۴). در یک تخلیه ادرار طبیعی، در ابتدا یک کاهش ارادی فشار داخل مجرای ادراری اتفاق می افتد که در نتیجه ریلکس شدن مجرا و عضلات مخطط اطراف آن می باشد. در ادامه، یک انقباض ممتد در عضله صاف مثانه (دترسور) اتفاق می افتد که این امر منجر به تخلیه مثانه در یک مدت زمان معلوم میگردد. بنابراین یک پروسه تخلیه طبیعی، یک جریان طبیعی و سریع و بدون حجم ادراری باقی مانده (PVR)بعد از تخلیه است که در نتیجه ریلکس شدن مجرای ادراری بدون افزایش فشار دترسور رخ میدهد.(۲) علل اختلال تخلیه ادرار بصورت کلی میتواند کاهش فعالیت یا از دست رفتن قابلیت انقباض دترسور یا انسداد خروجی مثانه ((BOO باشد(۱). مطابق با نتایج یک مطالعه آینده نگر، بیشتر افراد مبتلا به اختلال تخلیه ادرار، از دسته BOO هستند(۲). BOO اشاره به حالتی دارد که علیرغم وجود عفونت یا آسیب واضح، در حین تخلیه ادرار فشار دترسور افزایش و سرعت جریان ادرار کاهش بیابد. این حالت ممکن است همراه افزایش مقدار PVR باشد(۲, ۵). BOO در زنان به علل ساختاری یا عملکردی رخ میدهد. دلایل عمده BOO عملکردی، احتباس عملکردی (DV)و دیس سیرژی دریچه و دترسور((DSDمی باشد(۱). DV طبق تعریف انجمن بین المللی دفع، به جریان ادرار منقطع و/یا نوسان دار به علت انقباضات منقطع غیرارادی عضلات مخطط اطراف مجرای ادراری در فاز تخلیه ادرار، در افرادی که به لحاظ نورولوژیک سالم هستند، اطلاق میشود. DSD یک اختلال نورولوژیک است که طبق تعریف این انجمن به انقباض عضله دترسور که بصورت غیرارادی با انقباض عضلات مخطط مجرای ادراری و/یا اطراف مجرا همزمان شده است، اطلاق میشود. در چنین شرایطی، گاهی جریان ادرار کاملا قطع میشود (۵, ۶). اختلال DSD در بیماران نورولوژی با آسیب به طناب نخاعی که ارتباط بین ساقه مغزی و مراکز نخاعی ساکرال را تحت تاثیر قرار میدهد، دیده میشود (۷).

بیان مسئله

تشخیص BOO معمولا از طریق بررسی همزمان سرعت جریان ادرار و فشار دترسور در Pressure flow study میسر میشود(۲). در زنان، اگر شاخص انسداد خروجی مثانه (BOOI)بیشتر از ۱۸ باشد، اختلال تخلیه ادرار انسدادی تشخیص داده می شود. همچنین مقادیر بالای حجم باقی مانده ادراری (> ۵۰ میلی لیتر) و فرکانس ادراری بیش از حد در طی روز مهم ترین علائم قابل ارزیابی در این بیماران است(۸, ۹). بیشتر چالش این بیماران مسائل مربوط به مراجعه مکرر به سرویس بهداشتی از جمله عدم خواب مناسب در شب یا نگرانی مداوم در طول روز بابت این مسئله می باشد(۱۰). همچنین عفونت ادراری و بازگشت ادرار بصورت مزمن یا حاد که میتواند دردناک یا بدون درد باشد، از سایر مشکلاتی است که میتواند به دنبال این اختلال رخ دهد(۱).

تشخیص نهایی اختلال تخلیه ادرار براساس علائم بیمار و نتایج نهایی بررسی ها از جمله مطالعه یورودینامیک صورت میگیرد(۱). تاریخچه ادراری ۳ روزه، یوروفلومتری برای تشخیص الگوی تخلیه ادراری، استفاده از سونوگرافی برای تعیین میزان PVR، الکترومیوگرافی((EMGعضلات کف لگن برای افتراق میان انسداد ساختاری و عملکردی و اندوسکوپی برای تشخیص علت انسداد احتمالی از جمله تست های تشخیصی کلینیکی در اختلال تخلیه ادرار هستند(۱, ۱۱). حجم باقی مانده ادرار بعد از تخلیه و کارایی تخلیه مثانه ۲ متغیر بالینی هستند که متداولا برای ارزیابی بیماران مبتلا به اختلالات تخلیه ادرار از جمله اختلال تخلیه ادرار انسدادی به منظور بررسی اثر گذاری مداخله درمانی مورد استفاده قرار میگیرند (۱۲). مطالعات نشان داده اند که علائم

مرتبط با اختلال تخلیه ادرار به تنهایی شاخص های مورد اعتمادی برای تشخیص این دسته از اختلالات نیستند و شاخص های غیرطبیعی یوروفلومتری برای تشخیص این اختلالات و مطالعات سنجش فشار-جریان ادرار برای افتراق بین اختلال تخلیه ادرار به دنبال کاهش انقباض پذیری مثانه و اختلال تخلیه ادرار انسدادی نیاز است. جریان حداکثر ادرار کمتر از ۱۵ میلی لیتر بر ثانیه و حجم باقی مانده ادرار بعد از تخلیه بیشتر از ۵۰ میلی لیتر، شاخص های تشخیصی قابل اعتماد برای تشخیص اختلال تخلیه ادرار می باشد (۹). هدف اصلی از درمان اختلال تخلیه ادرار، نرمال کردن الگوی تخلیه ادرار و پیشگیری از عوارض جانبی است.
فیزیوتراپی کف لگن و بیوفیدبک خط اول درمان اختلال تخلیه ادرار عملکردی هستند. سایر درمان ها میتواند شامل درمان های روانشناختی در بیمارانی که اختلالات روانی را نشان میدهند، درمان های دارویی مثل داروهای مهارکننده آلفا، تزریق بوتاکس در اسفنکتر خارجی و gamma-aminobutyric acid receptor agonists باشد، هر چند که این درمان ها از سطح شواهد بالینی بالایی برخوردار نیستند.(۲) استفاده متناوب از سونداژ متناوب (CIC)و استفاده از جریان های الکتریکی بصورت تهاجمی یا غیر تهاجمی نیز برحسب نوع اختلال تخلیه ادرار کاربرد دارند(۱).
جریان های الکتریکی تعدیل کننده عصبی، جریان هایی هستند که در اختلالات راه های تحتانی ادراری با هدف تاثیرگذاری بر اعصابی که عملکرد این سیستم را کنترل میکنند، کاربرد دارند. این جریان ها شامل استفاده از جریان های الکتریکی کف لگن با استفاده از الکترود های واژینال، مقعدی یا سطحی، جریان اینترفرنشیال، جریان مگنت، تحریک عصب تیبیال از راه پوست (PTNS)و تحریک عصب ساکرال ((SNS می باشند (۱۳). مطالعات نشان داده اند که جریان های الکتریکی از طریق افزایش ظرفیت عملکردی مثانه، ایجاد ریلکسیشن در عضلات کف لگن و کاهش فشار مثانه در اختلالات متفاوت مسیرهای تحتانی ادراری اثرگذار خواهند بود.(۱۴) مکانیسم عمل این جریان ها در بیماران با اختلال تخلیه ادرار از طریق افزایش فعالیت آوران هایی است که از اسفنکتر مجرای ادراری به نخاع میروند و در نتیجه موجب بهبود حس مثانه و کاهش مهار عضله دترسور مثانه میشود(۱۵). همچنین جریان های الکتریکی منجر به ایجاد مهار رفلکسی عصب لگنی و در نتیجه افزایش ظرفیت مثانه میشود. جریان های الکتریکی با تحریک آوران های عصب پودندال باعث فعال شدن وابران های عصب هایپوگاستریک میشود که در نتیجه موجب کاهش فعالیت سمپاتیک و کاهش انقباضات غیرارادی عضلات کف لگن میشود. از آنجایی که عضلات کف لگن نقش مهمی در رفلکس های ساکرال دارد، تحریک این ناحیه با جریان اینترفرنشیال کم فرکانس باعث فعال شدن وابران های کف لگن و تاثیر بر عملکرد مثانه در سطح ساکرال میشود. همچنین انقباضات متناوب عضلات کف لگن منجر به ایجاد هماهنگی در عملکرد تخلیه مثانه میشود (۱۶). جریان اینترفرنشیال شامل دو جریان سینوسی غیرهم فاز با فرکانس متوسط (۱-۱۰kHz) می باشد. این دو جریان بعلت فرکانس بالا میتوانند مقاومت پوست را کاهش داده و در بافت های عمقی تر، یک جریان تداخلی با فرکانس پایین و شدت متناوب، با تاثیرات مشابه این دسته از جریان ها را در بافت هدف ایجاد کنند (۱۷). مدت زمان استفاده از این جریان در هر جلسه بسته به نوع ضایعه میتواند متفاوت باشد. به هرحال اگر ضایعه حاد نباشد، ۳۰-۲۰ دقیقه بافت هدف باید در معرض این جریان قرار بگیرد (۱۸).
علیرغم انجام مطالعات بالینی متعدد بر روی اثرگذاری جریان اینترفرنشیال در اختلالات متنوع کف لگن از جمله بی اختیاری ادراری و یبوست (۱۳, ۱۹, ۲۰)، مطابق با روش جست و جوی انجام گرفته، مطالعه ای پیرامون اثر گذاری این جریان بر علائم اختلالات تخلیه ادراری در زنان صورت نگرفته است. هرچند اخیرا مطالعاتی پیرامون تاثیر گذاری این جریان در کودکان مبتلا به اختلالات تخلیه ادراری صورت گرفته است (۱۴, ۲۱).
بنابراین هدف از مطالعه پیش رو، بررسی اثر گذاری جریان اینترفرنشیال بر علائم ادراری تحتانی و یافته های یوروفلومتری در زنان مبتلا به اختلال تخلیه ادرار می باشد. به این منظور، این بیماران به صورت تصادفی در ۲ گروه درمان و کنترل دسته بندی میشوند. درمان پایه در هر ۲ گروه شامل دارودرمانی، یوروتراپی روتین و تمرین درمانی اختصاصی اختلال تخلیه ادرار عملکردی خواهد بود. یورتراپی روتین عموما شامل توضیح ساده و مختصر درمورد عملکرد راه های ادراری و دفعی، تغذیه، مصرف مناسب مایعات، برنامه مناسب تخلیه ادرار بصورت ۲-۳ ساعت یکبار و آموزش شیوه مناسب استفاده از سرویس بهداشتی میشود. گروه

درمان ۱۰ جلسه جریان الکتریکی اینترفرنشیال واقعی با الکترودگذاری و اصول استاندارد و گروه کنترل ۱۰ جلسه جریان الکتریکی اینترفرنشیال بصورت شم دریافت میکنند. در انتهای درمان با مقایسه نتایج معاینات بالینی و تست های یوروفلومتری و باقی مانده ادراری ((PVRدر سونوگرافی در ۲ گروه، میتوان میزان اثر بخشی این جریان در این دسته از بیماران را اندازه گیری کرد. در صورتیکه اثربخشی مناسب جریان اینترفرنشیال در زنان مبتلا به اختلال تخلیه ادرار انسدادی عملکردی به اثبات برسد، در آینده میتواند به عنوان یکی از شیوه های درمانی غیرتهاجمی مورد استفاده قرار بگیرد.

خلاصه ضرورت اجرای طرح

با توجه به بهره گیری از جریان الکتریکی اینترفرنشیال به عنوان یک جریان تعدیل کننده راه های عصبی در اختلالات متنوع کف لگن از جمله بی اختیاری های ادراری، مبانی تئوری، استفاده از این جریان را بر اساس افزایش فعالیت آوران های اسفنکتر مجرای ادراری و ایجاد ریلکسیشن در عضلات کف لگن در اختلال تخلیه ادرار حمایت می کنند (۱, ۲). علی رغم وجود مطالعات هر چند محدود در زمینه استفاده از جریان اینترفرنشیال در اختلال تخلیه ادرار در جمعیت کودکان، مطالعه ای در این حیطه در جمعیت زنان مبتلا به این اختلال یافت نشد (۱, ۳). با توجه به اثر گذاری روی کیفیت زندگی و احتمال ایجاد عفونت و درد های مزمن درصورت عدم درمان (۴)، لزوم بررسی تاثیر جریان اینترفرنشیال در زنان مبتلا به اختلال تخلیه ادرار عملکردی به عنوان یک گزینه درمانی غیر تهاجمی به روشنی معلوم می باشد.

خلاصه روش اجرا و شیوه های تحلیل

روش کار:
پس از تصویب پروژه، گرفتن کد اخلاق نمونه گیری را با روش غیراحتمالی دردسترس انجام خواهد شد. به این صورت که پزشک متخصص اورولوژی بیماران مبتلا به اختلال تخلیه ادرار را معاینه و ارزیابی خواهند کرد. معاینات پزشک متخصص شامل گرفتن تاریخچه و پرسش درمورد علائم بالینی فرد، ارزیابی کامل بیمار به لحاظ درگیری های نورولوژی، انواع اختلالات اورولوژی، سیستومتری (به منظور سنجش فشار دترسور در فاز پر شدن و فشار جریان ادرار در فاز تخلیه مثانه(۶)) و تست های یورودینامیک و سونوگرافی با مثانه پر و خالی، به منظور افتراق نوع اختلال تخلیه ادراری خواهد بود. ارزیابی نورولوژی شامل بررسی تون ناحیه آنال، کنترل ارادی دریچه آنال، بررسی رفلکس های ناحیه تناسلی و رفلکس های اندام تحتانی و بررسی حس ناحیه تناسلی می شود. سپس درصورتی که بیمار مراجعه کننده، با معیار های ورود مطالعه پیش رو همخوانی داشته باشد، مطابق با تشخیص پزشک متخصص وارد این طرح خواهند شد. در ابتدا به بیماران تاریخچه ادراری ۳ روزه را داده و طریقه تکمیل آن را آموزش داده خواهد شد. پزشک متخصص از بیمار می خواهند که از روز بعد ویزیت به مدت ۳ روز این برگه را به دقت تکمیل کرده و مقادیر مایعات مصرفی و نوع مایعات مصرفی و دفعات مراجعه به سرویس بهداشتی را به صورت کامل در طی ۳ روز ثبت کنند. سپس به بیمار مقدمه ای از درمان پیش رو توضیح داده خواهد شد و ایشان را به همراه پرونده کامل مدارک پزشکی که شامل تست یورودینامیک و سونوگرافی می باشد، برای ۳ روز آینده به کلینیک های توانبخشی دانشگاه علوم پزشکی ایران ارجاع خواهند داد. پزشک متخصص اورولوژی همکار در طرح پیش رو به عنوان ارزیابی کننده پیامد های یورودینامیک نسبت به گروه بندی بیماران که در ادامه انجام خواهد گرفت، بی اطلاع خواهد ماند.
در کلینیک فیزیوتراپی، در ابتدا به تمام افراد توضیح جامعی از روند کامل کار و هدف از انجام تحقیق داده خواهد شد و سپس از همه آن ها رضایت نامه شخصی اخذ خواهد شد. به بیماران توضیح داده خواهد شد که ۲ گروه درمانی وجود دارد که همه درمان

ها در هر ۲ گروه به جز نحوه اعمال جریان الکتریکی اینترفرنشیال مشابه هستند و گروه بندی به صورت کاملا تصادفی صورت خواهد گرفت و امکان قرار گرفتن آنها در هر کدام از گروه ها با احتمال یکسان وجود دارد. همچنین به آنها این اطمینان داده خواهد شد که اگر در انتهای پروژه تغییر معناداری در پیامدهای تحقیق دیده شد، درمان انتخابی برای گروهی که آنرا دریافت نکردند به صورت کامل انجام خواهد گرفت.
تاریخچه ادراری ۳ روزه تکمیل شده را از بیمار تحویل گرفته خواهد شد و از بین اطلاعات موجود در آن، فرکانس مراجعه به سرویس بهداشتی در ۲۴ ساعت استخراج خواهد شد. سپس با استفاده از لیست کامپیوتری شماره های تصادفی، بیماران به ۲ گروه تصادفی درمان و کنترل، با نسبت ۱:۱ با بلوک های ۴ تایی تقسیم خواهند شد. در ادامه پرسشنامه خودساخته جهت جمع آوری اطلاعات فردی هر بیمار شامل سن، قد و وزن در ابتدای جلسه ای اول هر ۲ گروه تکمیل خواهد شد. پس از آن به همه بیماران نسخه فارسی پرسشنامه ICIQ-FLUTS (۲۵) و PFDI-۲۰ (۳۹) داده خواهد شد تا بیماران با توجه به آن به ترتیب، به شدت علائم اداری تحتانی و شدت علائم کف لگن خود نمره بدهند.
در جلسه آخر درمان، مجددا نسخه فارسی پرسشنامه ICIQ-FLUTS به بیماران داده میشود تا نمره دهی آنها به شدت علائم ادراری تحتانی با قبل از شروع درمان مورد مقایسه قرار بگیرد. همچنین در این جلسه به بیماران نسخه فارسی پرسشنامه تغییرات عمومی بیمار (PGI-C) داده میشود تا از دید خودشان به تغییرات کلی وضعیت خود بعد از درمان نمره دهی کنند. درنهایت، از بیمار خواسته میشود تا تاریخچه ادراری ۳ روزه را دوباره تکمیل کرده و بعد از گذشت ۲ هفته از اتمام درمان، جهت انجام مجدد تست یوروفلومتری و معاینه و ارزیابی علائم بالینی همراه با برگه تکمیل شده تاریخچه ادراری ۳ روزه به پزشک متخصص اورولوژی مراجعه کند. همچنین یک دوره پیگیری ۳ ماهه در این مطالعه در نظر گرفته شده است، که بعد از گذشت این دوره یک بار دیگر نیز انجام تست یوروفلومتری تکرار خواهد شد و علائم بالینی بیماران بررسی میشود تا تاثیر طولانی مدت یوروتراپی روتین به تنهایی و یوروتراپی روتین به همراه جریان اینترفرنشیال در علائم بالینی و یافته های یوروفلومتری بیماران مبتلا به اختلال تخلیه ادرار انسدادی عملکردی نیز مورد بررسی قرار بگیرد.

کورسازی: در مطالعه پیش رو بیماران و پزشک متخصص ارزیابی کننده نتایج درمان نسبت به گروه بندی کور خواهند بود.
به منظور جلوگیری از اثر پلاسبوی جریان الکتریکی اینترفرنشیال و خطای انتخاب از جریان شم و کورسازی بیماران نسبت به گروه بندی استفاده خواهد شد (۴۴, ۴۵). روش های متنوعی به این منظور در مطالعات پیشین استفاده شده است. مطالعات جدید علاوه بر روش الکترود گذاری یکسان در هر ۲ گروه، از اعمال جریان اینترفرنشیال در بازه کوتاهی از درمان و سپس قطع کردن جریان استفاده کرده اند (۳۱, ۴۴). در مطالعه پیش رو، جهت ایجاد کورسازی بیماران نسبت به نوع جریان مورد استفاده از روش الکترود گذاری یکسان در هر دو گروه و همچنین اعمال جریان الکتریکی اینترفرنشیال برای ۱ دقیقه ابتدایی درمان در گروه کنترل شده خواهد شد. به منظور کاهش حداکثری اثرات درمانی این مدالیته، شدت جریان تا احساس ابتدایی بیماران گروه کنترل تنظیم خواهد شد؛ اما به گروه درمان جریان با شدتی در سطح قابل تحمل فرد اعمال خواهد شد.
به منظور پیشگیری از خطای ارزیابی از کورسازی متخصص ارزیابی کننده بیماران استفاده خواهد شد (۴۵). به این منظور گروه بندی بیماران بعد از ارجاع آنها از سمت پزشک متخصص مربوطه صورت خواهد گرفت و ایشان نسبت به گروه بندی بیماران اطلاع نخواهند داشت.
با توجه به ماهیت درمان مدنظر در این مطالعه، کورسازی فیزیوتراپیست مسئول درمان امکان پذیر نخواهد بود.
پنهان سازی تخصیص: روش هایی که به منظور پنهان سازی توالی گروه بندی از افراد شرکت کننده در کارآزمایی های بالینی با هدف کاهش خطای انتخاب به کار برده میشود، پنهان سازی تخصیص نامیده می شود . در این مطالعه به منظوری پنهان سازی تخصیص از پاکت نامه های کدر مهر و موم شده که در آن گروه بندی ها با بلوک های ۴ تایی قرار میگیرند، استفاده خواهد شد. آماده سازی پاکت نامه ها و همچنین انتخاب یکی از آنها بعد از پذیرش بیمار، توسط افراد مستقل خارج از مطالعه صورت خواهد

گرفت. بنابراین تا قبل از باز شدن پاکت نامه، بیماران و درمانگران حاضر در مطالعه از چگونگی توالی گروه بندی اطلاعی نخواهند داشت (۴۶).

شیوه های تحلیل:در ابتدا شاخص‌های توصیفی شامل میانگین و انحراف معیار متغیرهای کمی و فراوانی و درصد متغیرهای کیفی مورد مطالعه در هر گروه، قبل و بعد از مداخله و بعد از دوره پیگیری ۳ ماهه گزارش می‌گردد. جهت بررسی نرمال بودن داده ها در گروه‌های مورد مطالعه از آزمون شاپیرو-ویلک استفاده می‌شود. جهت مقایسه شاخص های توصیفی کمی در دو گروه و در صورت نرمال بودن داده‌ها، از آزمون t مستقل دو نمونه‌ای و در غیر اینصورت از معادل ناپارامتری (آزمون من-ویتنی) استفاده می‌شود. جهت مقایسه شاخص های توصیفی کیفی در دو گروه از آزمون کای دو استفاده می شود. جهت بررسی و مقایسه میانگین میانگین PVR، حداکثر جریان ادرار ((Qmax، مدت زمان تخلیه ادرار، کارایی تخلیه مثانه، شدت علائم ادراری تحتانی و فرکانس مراجعه به سرویس بهداشتی در ۲۴ ساعت براساس تاریخچه ادراری ۳ روزه در زنان مبتلا به اختلال تخلیه ادرار در دو گروه مورد مطالعه در قبل و بعد از مداخله (درمان) و بعد از دوره پیگیری ۳ ماهه، در صورت نرمال بودن داده ها از آزمون آماری تحلیل واریانس اندازه‌های تکراری و در صورت عدم نرمال بودن از آزمون ناپارامتری معادل (آزمون فریدمن) استفاده خواهد شد. به منظور مقایسه میانگین شاخص‌های ذکر شده در فوق در دو گروه به تفکیک در هر دوره زمانی (قبل از مداخله، بعد از مداخله و بعد از دوره پیگیری سه ماهه) نیز در صورت نرمال بودن داده ها از آزمون t مستقل دو نمونه ای و در غیر اینصورت از معادل ناپارامتری آن (آزمون من-ویتنی) استفاده می شود. به منظور مقایسه فراوانی الگوی طبیعی تخلیه ادرار زنان مبتلا به اختلال تخلیه ادرار انسدادی عملکردی در قبل و بعد از مداخله (درمان) و بعد از دوره پیگیری ۳ ماهه به تفکیک در هر گروه مورد مطالعه از آزمون Q-کوکران استفاده می‌گردد. پس از جمع‌آوری اطلاعات، داده‌ها با استفاده از نرم افزار SPSS نسخه ۲۶ تحلیل خواهند شد. سطح معنی‌داری در این مطالعه ۵ درصد در نظر گرفته شده است.

اهداف کلی , اختصاصی و کاربردی

هدف کلی

تعیین و مقایسه اثرات جریان اینترفرنشیال و درمان یوروتراپی روتین بر علائم بالینی و یافته های یورودینامیک در زنان مبتلا به اختلال تخلیه ادرار

اهداف جزئی

اهداف توصیفی

تعیین میانگین (انحراف معیار) PVR، قبل و بعد از درمان در گروه جریان الکتریکی اینترفرنشیال واقعی

تعیین میانگین (انحراف معیار) PVR، قبل و بعد از درمان در گروه جریان الکتریکی اینترفرنشیال شم

تعیین میانگین (انحراف معیار) حداکثر جریان ادرار ((Qmax، قبل و بعد از درمان در گروه جریان الکتریکی اینترفرنشیال واقعی

تعیین میانگین (انحراف معیار) حداکثر جریان ادرار ((Qmax، قبل و بعد از درمان در گروه جریان الکتریکی اینترفرنشیال شم

تعیین میانگین (انحراف معیار) مدت زمان تخلیه ادرار، قبل و بعد از درمان در گروه جریان الکتریکی اینترفرنشیال واقعی

تعیین میانگین (انحراف معیار) مدت زمان تخلیه ادرار، قبل و بعد از درمان در گروه جریان الکتریکی اینترفرنشیال شم

تعیین میانگین (انحراف معیار) کارایی تخلیه مثانه، قبل و بعد از درمان در گروه الکتریکی اینترفرنشیال واقعی

تعیین میانگین (انحراف معیار) کارایی تخلیه مثانه، قبل و بعد از درمان در گروه جریان الکتریکی اینترفرنشیال شم

تعیین فراوانی الگوی طبیعی تخلیه ادرار، قبل و بعد از درمان در گروه جریان الکتریکی اینترفرنشیال واقعی

تعیین فراوانی الگوی طبیعی تخلیه ادرار، قبل و بعد از درمان در گروه جریان الکتریکی اینترفرنشیال شم

تعیین میانگین (انحراف معیار) فرکانس مراجعه به سرویس بهداشتی در ۲۴ ساعت براساس تاریخچه ادراری ۳ روزه، قبل و بعد از درمان در گروه جریان الکتریکی اینترفرنشیال واقعی

تعیین میانگین (انحراف معیار) فرکانس مراجعه به سرویس بهداشتی در ۲۴ ساعت براساس تاریخچه ادراری ۳ روزه، قبل و بعد از درمان در گروه جریان الکتریکی اینترفرنشیال شم

تعیین میانگین (انحراف معیار) شدت علائم ادراری تحتانی، قبل و بعد از درمان در گروه جریان الکتریکی اینترفرنشیال واقعی

تعیین میانگین (انحراف معیار) شدت علائم ادراری تحتانی، قبل و بعد از درمان در گروه جریان الکتریکی اینترفرنشیال شم

تعیین میانگین میزان تغییرات پس از درمان از نگاه بیمار ((PGICدر گروه جریان الکتریکی اینترفرنشیال واقعی

تعیین میانگین میزان تغییرات پس از درمان از نگاه بیمار((PGICدر گروه جریان الکتریکی اینترفرنشیال شم

اهداف تحلیلی:

مقایسه میانگین تغییرات PVR، قبل و بعد درمان و بعد از دوره پیگیری ۳ ماهه در گروه جریان الکتریکی اینترفرنشیال شم و جریان الکتریکی اینترفرنشیال واقعی

مقایسه میانگین تغییرات حداکثر جریان ادرار ((Qmax، قبل و بعد درمان و بعد از دوره پیگیری ۳ ماهه در گروه جریان الکتریکی اینترفرنشیال شم و جریان الکتریکی اینترفرنشیال واقعی

مقایسه میانگین مدت زمان تخلیه ادرار، قبل و بعد درمان و بعد از دوره پیگیری ۳ ماهه در گروه جریان الکتریکی اینترفرنشیال شم و جریان الکتریکی اینترفرنشیال واقعی

مقایسه میانگین تغییرات فرکانس استفاده از سرویس بهداشتی در ۲۴ ساعت براساس تاریخچه ادراری ۳ روزه، قبل و بعد درمان و بعد از دوره پیگیری ۳ ماهه در گروه جریان الکتریکی اینترفرنشیال شم و جریان الکتریکی اینترفرنشیال واقعی

مقایسه میانه الگوی طبیعی تخلیه ادرار، قبل و بعد درمان و بعد از دوره پیگیری ۳ ماهه در گروه جریان الکتریکی اینترفرنشیال شم و جریان الکتریکی اینترفرنشیال واقعی

مقایسه میانگین شدت علائم ادراری تحتانی، قبل و بعد درمان و بعد از دوره پیگیری ۳ ماهه در گروه جریان الکتریکی اینترفرنشیال شم و جریان الکتریکی اینترفرنشیال واقعی

مقایسه میانگین تغییرات PVR، بعد درمان و بعد از دوره پیگیری ۳ ماهه، بین دو گروه جریان الکتریکی اینترفرنشیال شم و جریان الکتریکی اینترفرنشیال واقعی

مقایسه میانگین تغییرات حداکثر جریان ادرار ((Qmax، بعد درمان و بعد از دوره پیگیری ۳ ماهه، بین دو گروه جریان الکتریکی اینترفرنشیال شم و جریان الکتریکی اینترفرنشیال واقعی

مقایسه میانگین مدت زمان تخلیه ادرار، بعد درمان و بعد از دوره پیگیری ۳ ماهه، بین دو گروه جریان الکتریکی اینترفرنشیال شم و جریان الکتریکی اینترفرنشیال واقعی

مقایسه میانگین کارایی تخلیه ادرار، بعد درمان و بعد از دوره پیگیری ۳ ماهه، بین دو گروه جریان الکتریکی اینترفرنشیال شم و جریان الکتریکی اینترفرنشیال واقعی

مقایسه فراوانی الگوی طبیعی تخلیه ادرار، بعد درمان و بعد از دوره پیگیری ۳ ماهه، بین دو گروه جریان الکتریکی اینترفرنشیال شم و جریان الکتریکی اینترفرنشیال واقعی

مقایسه میانگین تغییرات فرکانس استفاده از سرویس بهداشتی در ۲۴ ساعت براساس تاریخچه ادراری ۳ روزه، بعد درمان و بعد از دوره پیگیری ۳ ماهه، بین دو گروه جریان الکتریکی اینترفرنشیال شم و جریان الکتریکی اینترفرنشیال واقعی

مقایسه میانگین شدت علائم ادراری تحتانی، بعد درمان و بعد از دوره پیگیری ۳ ماهه، بین دو گروه جریان الکتریکی اینترفرنشیال شم و جریان الکتریکی اینترفرنشیال واقعی

مقایسه میانگین میزان تغییرات پس از درمان از نگاه بیمار، بعد درمان و بعد از دوره پیگیری ۳ ماهه، بین دو گروه جریان الکتریکی اینترفرنشیال شم و جریان الکتریکی اینترفرنشیال واقعی

اهداف کاربردی:
در صورتی که درمان با جریان اینترفرشیال در زنان مبتلا به اختلال تخلیه ادرار، بتواند بهبود معناداری در علائم کلینیکی و یافته های یوروفلومتری این افراد ایجاد کند، در آینده این دسته از بیماران میتوانند برای دریافت این درمان جزو مراجعین کلینیک های فیزیوتراپی کف لگن قرار بگیرند.

سوالات پژوهشی و فرضیات

میانگین میزان PVR قبل و بعد از درمان در گروه جریان الکتریکی اینترفرنشیال واقعی تفاوت معناداری ندارد.
میانگین حداکثر جریان ادرار (Qmax)قبل و بعد از درمان در گروه جریان الکتریکی اینترفرنشیال واقعی تفاوت معناداری ندارد.
میانگین مدت زمان تخلیه ادرار قبل و بعد از درمان در گروه جریان الکتریکی اینترفرنشیال واقعی تفاوت معناداری ندارد.
میانگین کارایی تخلیه ادرار قبل و بعد از درمان در گروه جریان الکتریکی اینترفرنشیال واقعی تفاوت معناداری ندارد.
فراوانی الگوی طبیعی تخلیه ادرار قبل و بعد از درمان در گروه جریان الکتریکی اینترفرنشیال واقعی تفاوت معناداری ندارد.
میانگین فرکانس استفاده از سرویس بهداشتی در ۲۴ ساعت براساس تاریخچه ادراری ۳ روزه، قبل و بعد از درمان در گروه جریان الکتریکی اینترفرنشیال واقعی تفاوت معناداری ندارد.
میانگین نمرات نسخه فارسی پرسشنامه ICIQ-FLUTS (۲۵) قبل و بعد از درمان در گروه جریان الکتریکی اینترفرنشیال واقعی تفاوت معناداری ندارد.
میانگین میزان PVR بعد از درمان در گروه جریان الکتریکی اینترفرنشیال واقعی و گروه جریان الکتریکی اینترفرنشیال شم، تفاوت معناداری ندارد.
میانگین حداکثر جریان ادرار (Qmax)بعد از درمان در گروه جریان الکتریکی اینترفرنشیال واقعی و گروه جریان الکتریکی اینترفرنشیال شم، تفاوت معناداری ندارد.
میانگین مدت زمان تخلیه ادرار بعد از درمان در گروه جریان الکتریکی اینترفرنشیال واقعی و جریان الکتریکی اینترفرنشیال شم، تفاوت معناداری ندارد.
میانگین کارایی تخلیه مثانه بعد از درمان در گروه جریان الکتریکی اینترفرنشیال واقعی و گروه جریان الکتریکی اینترفرنشیال شم، تفاوت معناداری ندارد.
فراوانی الگوی طبیعی تخلیه ادرار بعد از درمان در گروه جریان الکتریکی اینترفرنشیال واقعی و گروه جریان الکتریکی اینترفرنشیال شم، تفاوت معناداری ندارد.
میانگین فرکانس استفاده از سرویس بهداشتی در ۲۴ ساعت براساس تاریخچه ادراری ۳ روزه، بعد از در گروه درمان جریان الکتریکی اینترفرنشیال واقعی وگروه جریان الکتریکی اینترفرنشیال شم، تفاوت معناداری ندارد.
میانگین نمرات نسخه فارسی پرسشنامه ICIQ-FLUTS بعد از درمان در گروه جریان الکتریکی اینترفرنشیال واقعی و گروه جریان الکتریکی اینترفرنشیال شم، تفاوت معناداری ندارد.
میانگین نمره پرسشنامه مقیاس کلی رتبه بندی تغییر(GRCS)در گروه درمان جریان الکتریکی اینترفرنشیال واقعی و جریان الکتریکی اینترفرنشیال شم، تفاوت معناداری ندارد.

فهرست منابع مورد استفاده

۱. Abdel Raheem A, Madersbacher H. Voiding dysfunction in women: How to manage it correctly. Arab journal of urology. ۲۰۱۳;۱۱(۴):۳۱۹-۳۰.

۲. Artibani W, Cerruto MA. Dysfunctional voiding. Current opinion in urology. ۲۰۱۴;۲۴(۴):۳۳۰-۵.

۳. Yang TH, Chuang FC, Kuo HC. Urodynamic characteristics of detrusor underactivity in women with voiding dysfunction. PloS one. ۲۰۱۸;۱۳(۶):e۰۱۹۸۷۶۴.

۴. Chapple CR, Osman NI, Birder L, Dmochowski R, Drake MJ, van Koeveringe G, et al. Terminology report from the international continence society (ICS) working group on underactive bladder (UAB). ۲۰۱۸;۳۷(۸):۲۹۲۸-۳۱.

۵. Abrams P, Cardozo L, Fall M, Griffiths D, Rosier P, Ulmsten U, et al. The standardization of terminology of lower urinary tract function: report from the standardization sub-committee of International Continence Society. Textbook of Female Urology and Urogynecology: CRC Press; ۲۰۱۰. p. ۱۰۹۸-۱۰۸.

۶. Haylen BT, De Ridder D, Freeman RM, Swift SE, Berghmans B, Lee J, et al. An International Urogynecological Association (IUGA)/International Continence Society (ICS) joint report on the terminology for female pelvic floor dysfunction. ۲۰۱۰;۲۹(۱):۴-۲۰.

۷. Pfeiffer RF. CHAPTER ۲۹ - BLADDER AND SEXUAL FUNCTION AND DYSFUNCTION. In: Schapira AHV, Byrne E, DiMauro S, Frackowiak RSJ, Johnson RT, Mizuno Y, et al., editors. Neurology and Clinical Neuroscience. Philadelphia: Mosby; ۲۰۰۷. p. ۳۶۲-۷۱.

۸. Santis-Moya F, Calvo CI, Rojas T, Dell'Oro A, Baquedano P, Saavedra A. Urodynamic and clinical features in women with overactive bladder: When to suspect concomitant voiding dysfunction? Neurourology and urodynamics. ۲۰۲۱;۴۰(۶):۱۵۰۹-۱۴.

۹. Lemack GE. Urodynamic assessment of bladder-outlet obstruction in women. Nature clinical practice Urology. ۲۰۰۶;۳(۱):۳۸-۴۴.

۱۰. Uren AD, Drake MJ. Definition and symptoms of underactive bladder. Investigative and clinical urology. ۲۰۱۷;۵۸(Suppl ۲):S۶۱-s۷.

۱۱. Abrams P, Andersson K-E, Birder L, Brubaker L, Cardozo L, Chapple C, et al. Fourth International Consultation on Incontinence Recommendations of the International Scientific Committee: Evaluation and treatment of urinary incontinence, pelvic organ prolapse, and fecal incontinence. ۲۰۱۰;۲۹(۱):۲۱۳-۴۰.

۱۲. Yono M, Ito K, Oyama M, Tanaka T, Irie S, Matsukawa Y, et al. Variability of post-void residual urine volume and bladder voiding efficiency in patients with underactive bladder. Lower urinary tract symptoms. ۲۰۲۱;۱۳(۱):۵۱-۵.

۱۳. Yamanishi T, Kaga K, Fuse M, Shibata C, Uchiyama TJLLUTS. Neuromodulation for the treatment of lower urinary tract symptoms. ۲۰۱۵;۷(۳):۱۲۱-۳۲.

۱۴. Sharifi-Rad L, Seyedian S-SL, Fatemi-Behbahani S-M, Lotfi B, Kajbafzadeh A-MJJoPU.

Impact of transcutaneous interferential electrical stimulation for management of primary bladder neck dysfunction in children. ۲۰۲۰;۱۶(۱):e۳۶. e۱. -۶.

۱۵. Bosch JRJBi. Electrical neuromodulatory therapy in female voiding dysfunction. ۲۰۰۶;-۹۸:۴۳-۸.

۱۶. Sharifi-Rad L, Ladi-Seyedian S-S, Kajbafzadeh A-MJUJ. Interferential Electrical Stimulation Efficacy in the Management of Lower Urinary Tract Dysfunction in Children: A Review of the Literature. ۲۰۲۱;۱۸(۵).

۱۷. Rampazo É P, Liebano RE. Analgesic Effects of Interferential Current Therapy: A Narrative Review. Medicina (Kaunas, Lithuania). ۲۰۲۲;۵۸(۱).

۱۸. Watson T. Interferential Therapy (IFT). ۲۰۱۵.

۱۹. Vitton V, Mion F, Leroi AM, Brochard C, Coffin B, Zerbib F, et al. Interferential therapy for chronic constipation in adults: The CON-COUR randomizedcontrolled trial. United European gastroenterology journal. ۲۰۲۳;۱۱(۴):۳۳۷-۴۹.

۲۰. Southwell BR. Medical devices to deliver transcutaneous electrical stimulation using interferential current to treat constipation. Expert review of medical devices. ۲۰۱۳;۱۰(۶):۷۰۱-۴.

۲۱. Kajbafzadeh AM, Sharifi-Rad L, Ladi-Seyedian SS, Mozafarpour SJBi. Transcutaneous interferential electrical stimulation for the management of non-neuropathic underactive bladder in children: a randomised clinical trial. ۲۰۱۶;۱۱۷(۵):۷۹۳-۸۰۰.

۲۲. Ladi-Seyedian SS, Sharifi-Rad L, Kajbafzadeh AM. Management of Bladder Bowel Dysfunction in Children by Pelvic Floor Interferential Electrical Stimulation and Muscle Exercises: A Randomized Clinical Trial. Urology. ۲۰۲۰;۱۴۴:۱۸۲-۷.

۲۳. Moore JS, Gibson PR, Burgell RE. Randomised clinical trial: transabdominal interferential electrical stimulation vs sham stimulation in women with functional constipation. Alimentary pharmacology & therapeutics. ۲۰۲۰;۵۱(۸):۷۶۰-۹.

۲۴. Pauwels N, Willemse C, Hellemans S, Komen N, Van den Broeck S, Roenen J, et al. The role of neuromodulation in chronic functional constipation: a systematic review. Acta gastro-enterologica Belgica. ۲۰۲۱;۸۴(۳):۴۶۷-۷۶.

۲۵. Pourmomeny AA, Rezaeian ZS, Soltanmohamadi M. Translation and linguistic validation of the Persian version of the Bristol Female Lower Urinary Tract Symptoms instrument. International urogynecology journal. ۲۰۱۷;۲۸(۹):۱۳۲۹-۳۳.

۲۶. Schulman SL, Quinn CK, Plachter N, Kodman-Jones CJP. Comprehensive management of dysfunctional voiding. ۱۹۹۹;۱۰۳(۳):e۳۱-e.

۲۷. Abrams P, Cardozo L, Fall M, Griffiths D, Rosier P, Ulmsten U, et al. The standardisation of terminology in lower urinary tract function: report from the standardisation sub-committee of the International Continence Society. ۲۰۰۳;۶۱(۱):۳۷-۴۹.

۲۸. Jin X, Tang H, Chen GJU. CT three-dimensional visualization model in diagnosis and treatment of stress urinary incontinence: A retrospective study. ۲۰۲۳;۱۷۲:۸۴-۸.

۲۹. Rohr G, Christensen K, Ulstrup K, Kragstrup JJAoegS. Reproducibility and validity of

.۹۶۹-۷۲):۱۰(۸۳;۲۰۰۴simple questions to identify urinary incontinence in elderly women.

Nieuwhof-Leppink AJ, Hussong J, Chase J, Larsson J, Renson C, Hoebeke P, et al. .۳۰
Definitions, indications and practice of urotherapy in children and adolescents: - A standardization document of the International Children's Continence Society (ICCS). Journal of .۱۷۲-۸۱):۲(۱۷;۲۰۲۱pediatric urology.

Rakel B, Cooper N, Adams HJ, Messer BR, Frey Law LA, Dannen DR, et al. A new .۳۱
transient sham TENS device allows for investigator blinding while delivering a true placebo .۲۳۰-۸):۳(۱۱;۲۰۱۰treatment. The journal of pain.

Abrams P. Bladder outlet obstruction index, bladder contractility index and bladder voiding .۳۲
efficiency: three simple indices to define bladder voiding function. BJU international. .۱۴-۵):۱(۸۴;۱۹۹۹

.۱۸:۴۶۱-۵;۲۰۰۷Barber MDJIUJ. Questionnaires for women with pelvic floor disorders. .۳۳

Kim M, Jeong CW, Oh SJ. Diagnostic value of urodynamic bladder outlet obstruction to .۳۴
select patients for transurethral surgery of the prostate: Systematic review and meta-analysis. .۰۱۷۲۵۹۰):e۲(۱۲;۲۰۱۷PloS one.

Klingele CJ, Lightner DJ, Fletcher J, Gebhart JB, Bharucha AEJN, Motility. Dysfunctional .۳۵
.۲۸۴-e۱۰۹۴):۱۰(۲۲;۲۰۱۰urinary voiding in women with functional defecatory disorders.

Tayebi S, Salehi-Pourmehr H, Hajebrahimi S, Hashim H. Translation and validation of the .۳۶
.۳۲۸۷-۹۱):۱۲(۳۲;۲۰۲۱Persian ICIQ bladder diary. International urogynecology journal.

Lewis AL, Young GJ, Abrams P, Blair PS, Chapple C, Glazener CMA, et al. Clinical and .۳۷
Patient-reported Outcome Measures in Men Referred for Consideration of Surgery to Treat Lower Urinary Tract Symptoms: Baseline Results and Diagnostic Findings of the Urodynamics for Prostate Surgery Trial; Randomised Evaluation of Assessment Methods (UPSTREAM). .۳۴۰-۵۰):۳(۵;۲۰۱۹European urology focus.

https://iciq.net/iciq-bladder-diary. .۳۸

: ۲۰۸Hakimi S, Hajebrahimi S, Bastani P, Aminian E, Ghana S, Mohammadi MJBo. .۳۹
), Iranian ۲۰translation and validation of the pelvic floor distress inventory short form (PFDI-
.۲۰۱۶-۰۱۵۴۱۵,۲۰۸):bmjopen-۱(Suppl ۷;۲۰۱۷version.

Mashayekh-Amiri S, Asghari Jafarabadi M, Rashidi F, Mirghafourvand MJBWsH. .۴۰
Translation and measurement properties of the pelvic floor distress inventory-short form (PFDI- .۱-۱۱):۱(۲۳;۲۰۲۳) in Iranian reproductive age women. ۲۰

Sánchez Sánchez B, Torres Lacomba M, Navarro Brazález B, Cerezo Téllez E, Pacheco Da .۴۱
Costa S, Gutiérrez Ortega C. Responsiveness of the Spanish Pelvic Floor Distress Inventory and) in women with pelvic ۷and PFIQ- ۲۰Pelvic Floor Impact Questionnaires Short Forms (PFDI-
floor disorders. European journal of obstetrics, gynecology, and reproductive biology. .۱۹۰:۲۰-۵;۲۰۱۵

Bobos P, Ziebart C, Furtado R, Lu Z, MacDermid JC. Psychometric properties of the global .۴۲
rating of change scales in patients with low back pain, upper and lower extremity disorders. A .۲۱:۴۰-۸;۲۰۲۰systematic review with meta-analysis. Journal of orthopaedics.

۴۳. Lazaros T, Ioannis T, Vasileios S, Christina P, Michael S. The effect of pelvic floor muscle training in women with functional bladder outlet obstruction. Archives of gynecology and obstetrics. ۲۰۲۳;۳۰۷(۵):۱۴۸۹-۹۴.

۴۴. Mendonça Araújo F, Alves Menezes M, Martins de Araújo A, Abner dos Santos Sousa T, Vasconcelos Lima L, Ádan Nunes Carvalho E, et al. Validation of a New Placebo Interferential Current Method: A New Placebo Method of Electrostimulation. Pain Medicine. ۲۰۱۷;۱۸(۱):۸۶-۹۴.

۴۵. Day SJ, Altman DG. Statistics notes: blinding in clinical trials and other studies. BMJ (Clinical research ed). ۲۰۰۰;۳۲۱(۷۲۵۹):۵۰۴.

۴۶. Altman DG, Schulz KF. Statistics notes: Concealing treatment allocation in randomised trials. BMJ (Clinical research ed). ۲۰۰۱;۳۲۳(۷۳۱۰):۴۴۶-۷.

تعریف واژه ها

مفاهیم

اختلال تخلیه ادراری

تعریف شرحی: مجموعه علائمی که در فاز تخلیه یا پس از تخلیه ادرار تجربه میشوند؛ از جمله، جریان آرام ادرار، چند شاخه بودن یا پاشیدن ادرار، جریان ادرار منقطع، سختی در شروع ادرار، زور زدن برای تخلیه، احساس تخلیه ناکامل مثانه، طولانی شدن فاز پایانی تخلیه ادرار بصورت چکه چکه شدن ادرار، نیاز سریع به تخلیه مجدد ادرار، امکان ادرار در پوزیشن های خاص و چکه چکه کردن ادرار بعد از اتمام تخلیه ادرار(۸, ۱۵).

تعریف کاربردی: در این مطالعه بیماران مبتلا به اختلال تخلیه ادرار از طریق ارجاع پزشک متخصص اورولوژی وارد مطالعه خواهند شد.

اختلال تخلیه ادرار انسدادی

تعریف شرحی: به کاهش سرعت جریان ادرار(سرعت حداکثری جریان ادرار<۱۵میلی لیتر بر ثانیه) همراه با افزایش فشار دترسور(>۲۰ سانتی متر آب) در زمان تخلیه ادرار، در غیاب عفونت ها و پاتولوژی های واضح اطلاق میشود که با مطالعه همزمان Pressure flow study و یوروفلومتری تشخیص داده میشود. این اختلال ممکن است همراه با افزایش مقدار باقی مانده ادراری باشد(۶, ۸). در این حالت، فاز پر شدن مثانه معمولا طبیعی است؛ به این صورت که عضله دترسور بی ثباتی و انقباضی از خود نشان نمیدهد و بعد از پر شدن نیمی از حجم مثانه فرد احساس نیاز برای رفتن به سرویس بهداشتی را خواهد داشت، اما در فاز تخلیه فعالیت الکتریکی در عضلات کف لگن ثبت میشود و PVR بعد از تخلیه متغیر است (۲۶). مقدار شاخص انسداد خروجی مثانه (BOOI)بیشتر از ۱۸ به نفع تشخیص اختلال تخلیه ادرار انسدادی می باشد (۱۲).

تعریف کاربردی: این مطالعه بیماران مبتلا به اختلال تخلیه ادرار انسدادی عملکردی توسط ارجاع متخصص اورولوژی وارد مطالعه میشوند. معیارهای تشخیصی برای این اختلال شاخص انسداد خروجی مثانه، جریان حداکثر ادرار، مدت زمان تخلیه ادرار در تست یورودینامیک و باقی مانده ادرار بعد از تخلیه در سونوگرافی خواهد بود.

بی اختیاری ادراری:

تعریف شرحی: طبق تعریف ICS بی اختیاری ادرار به هرگونه شکایت از نشت غیرارادی ادرار که روی زندگی اجتماعی، بهداشت و کیفیت زندگی فرد اثر میگذارد، اطلاق میشود. بی اختیاری ادراری انواع متعددی دارد که شایع ترین آنها به شرح زیر است:

بی اختیاری ادراری استرسی: به معنای نشت غیرارادی ادرار به دنبال افزایش فشار داخل شکمی نظیر زور زدن یا عطسه و سرفه (۲۷, ۲۸)

بی اختیاری ادراری اورژانسی: به معنای نشت غیر ارادی ادرار که همراه یا بلافاصله بعد از احساس فوریت در تخلیه ادرار بوجود می آید.

بی اختیاری ادراری مرکب: به معنای شکایت از نشت غیر ارادی ادرار که همراه با احساس فوریت در تخلیه ادرار و همچنین نشت غیر ارادی در زمان افزایش فشار داخل شکمی باشد.(۲۷)

تعریف کاربردی: در این مطالعه بیماران برای تشخیص بی اختیاری ادراری، باید به حداقل یکی از سوالات پیش رو پاسخ مثبت بدهد: ۱) آیا در ۱ ماه اخیر نشت غیرارادی ادرار را به دنبال فعالیت های فیزیکی خاصی مثل بلند کردن اجسام، خندیدن، سرفه یا عطسه تجربه کرده اید؟ ۲) آیا در ۱ ماه اخیر، احساس اضطرار در تخلیه ادراری به نحوی که نتوانید این احساس را کنترل کنید، داشته اید؟(۲۹)

یوروتراپی

تعریف شرحی: به همه مداخلات غیر جراحی و غیر دارویی برای درمان اختلالات ادراری تحتانی که در کودکان و بزرگسالان با هدف نرمال کردن الگوی تخلیه ادرار و مدفوع و پیش گیری از اختلالات عملکردی بیشتر از طریق تمرین مکرر کاربرد دارد، اطلاق میشود(۳۰).

تعریف کاربردی: در این مطالعه یوروتراپی استاندارد شامل توضیح و آموزش ساده درمورد عملکرد راه های ادراری و دفعی، مصرف مناسب مایعات، برنامه مناسب تخلیه ادرار بصورت ۲-۳ ساعت یکبار، آموزش شیوه مناسب استفاده از سرویس بهداشتی و درصورت نیاز شیوه استفاده درست از سوند یکبارمصرف میشود(۱). در آموزش شیوه درست استفاده از سرویس بهداشتی، راستای صحیح ستون فقرات حین تخلیه ادرار اهمیت دارد. همچنین به بیماران آموزش داده می شود که در حین تخلیه ادرار به خوبی ران ها را از هم دور، عضلات شکم و کف لگن را ریکس کنند و جریان ادرار را در طول زمان تخلیه مثانه قوی نگه دارد. به همه بیماران توصیه میشود که مانند دفع ادرار، برنامه منظمی هم برای دفع مدفوع داشته باشند و از غذاهای با فیبر زیاد استفاده کنند. به عنوان یک تمرین هم به تمام بیماران آموزش داده میشود که برای ۱۰ ثانیه عضلات کف لگن خود را منقبض کنند و برای ۳۰ ثانیه بعدی کاملا عضلات این ناحیه را ریلکس کنند (۳).

جریان اینترفرنشیال

تعریف شرحی: یک جریان فرکانس پایین(۱-۱۰۰ هرتز) و با شدت متناوب که جهت مقابله با مقاومت پوستی و تاثیر گذاری در بافت های عمقی از تداخل دو جریان با فرکانس متوسط (۱-۱۰ کیلوهرتز) در عمق بافت ایجاد میشود (۱۷, ۱۹). برای اثرگذاری این جریان در شرایط غیرحاد، توصیه شده است که در هر جلسه، به مدت زمان ۳۰-۲۰ دقیقه جریان به بیمار اعمال شود (۲۰).

تعریف کاربردی: در این مطالعه از جریان اینترفرنشیال دستگاه استاندارد الکتروتراپی (نوین) با فرکانس حامل ۴۰۰۰ هرتز و فرکانس ضربان ۸۰-۱۶۰ هرتز به مدت ۳۰ دقیقه در ناحیه کف لگن استفاده خواهد شد. الکترود ها به صورت ضربدری در ناحیه سمفیزیس پوبیس و توبروزیتی های ایسکیال قرار می گیرند تا جریان از ناحیه کف لگن عبور کند. شدت جریان در گروه جریان الکتریکی اینترفرنشیال واقعی تا حداکثر تحمل بیمار زیاد می شود و در طی ۳۰ دقیقه درمان شدت با توجه به تطابق جریان با بدن تعدیل شده و قوی می ماند. در گروه جریان الکتریکی اینترفرنشیال شم به مدت ۱ دقیقه ابتدایی جریان الکتریکی برای بیماران وصل میشود و سپس جریان قطع میشود (۳۱).

متغیرها

شدت علائم ادراری تحتانی:

تعریف شرحی: نشانه های ذهنی یک بیماری که توسط بیمار، مراقب یا خانواده اش درک میشود و را به سمت پیدا کردن درمان

سوق میدهد. این دسته از نشانه ها معمولا در شرح حال گیری از بیمار به دست میآیند و معمولا کیفی هستند.(۲۷)

تعریف کاربردی: در این پژوهش به منظور بررسی شدت علائم ادراری تحتانی از نسخه فارسی دو پرسشنامه ICIQ-FLUTS استفاده میشود. در این پرسشنامه، سوالات در ۳ دسته علائم فاز پرشدن مثانه، علائم فاز تخلیه مثانه و علائم بی اختیاری ادراری تقسیم میشود. در این مطالعه علائم فاز تخلیه به عنوان متغیر وابسته و علائم بی اختیاری ادراری به عنوان متغیر مداخله گر در نظر گرفته می شود.

باقی مانده ادرار بعد از تخلیه ((PVR

تعریف شرحی: به باقی مانده ادرار در مثانه بعد از اتمام کامل تخلیه ادرار ساده و بدون استفاده از سوند اطلاق میشود (۱۰).

مشاهدات بالینی نشان داده اند که مقدار حجم باقی مانده ادرار بعد از تخلیه با ظرفیت مثانه یا حجم مثانه قبل از تخلیه ادرار ارتباط دارد؛ به این معنا که هرچه حجم ادرار موجود در مثانه قبل از تخلیه ادرار بیشتر باشد، مقدار حجم باقی مانده ادرار بعد از تخلیه نیز بیشتر خواهد بود (۱۶). با توجه به ورود مداوم ادرار داخل مثانه به صورت ۱۴-۱ میلی لیتر در ثانیه، اندازه گیری این متغیر باید با کمترین فاصله از اتمام تخلیه ادرار صورت بگیرد. انجام سونوگرافی این امکان را فراهم می آور که این اندازه گیری در سریع ترین زمان ممکن در حدود ۶۰ ثانیه بعد از اتمام تخلیه ادرار انجام بگیرد (۱۰). اگر مقدار PVR بیش تر از ۵۰ میلی لیتر باشد، به نفع تشخیص اختلال تخلیه ادرار است (۱۳).

تعریف کاربردی: در این مطالعه به منظور اندازه گیری حجم باقی مانده ادرار بعد از تخلیه، از انجام سونوگرافی با مثانه پر و خالی توسط پزشک متخصص اورولوژی استفاده خواهد شد (۱۳).

جریان حداکثر ادرار

تعریف شرحی: جریان حداکثر ادرار که با علامت Qmax در مطالعات نشان داده میشود به معنای بیشترین سرعت جریان ادرار اندازه گیری شده است و با واحد میلی لیتر بر ثانیه گزارش میشود (۱۰). به طور کلی در اختلالات تخلیه ادرار جریان حداکثر ادرار کاهش می یابد. مطالعات نشان داده اند که مقادیر کمتر از ۱۵ میلی لیتر بر ثانیه در زنان به نفع تشخیص اختلال تخلیه ادرار است (۱۳).

تعریف کاربردی: در این مطالعه جریان حداکثر ادرار به عنوان یک متغیر گزارش شده در تست یوروفلومتری که توسط پزشک متخصص اورولوژی انجام میشود، گزارش داده میشود.

کارایی تخلیه مثانه

تعریف شرحی: کارایی تخلیه مثانه ((BVE یک متغیر برای سنجش انقباض پذیری مثانه در برابر مقاومت مجرای ادراری می باشد. این متغیر مقدار حجم تخلیه شده مثانه را به صورت درصدی از ظرفیت کلی مثانه بیان می کند (۳۲). حجم کلی مثانه از طریق مجموع حجم باقی مانده ادرار بعد از تخلیه و حجم ادرار تخلیه شده بدست می آید (۱۶).

تعریف کاربردی: در این مطالعه این مولفه از طریق سونوگرافی با مثانه پر و خالی توسط پزشک متخصص اورولوژی و با استفاده از فرمول (حجم ادرار تخلیه شده / ظرفیت کلی مثانه)* ۱۰۰، بدست خواهد آمد(۳۲).

مدت زمان تخلیه ادرار

تعریف شرحی: به طول مدت زمان کلی تخلیه ادرار که میتواند شامل وقفه های متعدد نیز بشود، زمان تخلیه ادرار گفته میشود. در صورتی که منحنی تخلیه ادرار پیوسته و بدون وقفه باشد زمان تخلیه ادرار و زمان جریان ادرار یکسان خواهند بود (۱۰).

تعریف کاربردی: در این مطالعه مدت زمان تخلیه ادرار به عنوان یک متغیر گزارش شده در تست یوروفلومتری که توسط پزشک متخصص اورولوژی انجام میشود، گزارش داده خواهد شد.

الگوی تخلیه ادرار

تعریف شرحی: در تست یوروفلومتری فرد ادرار خود را دریک مخزن که قابلیت سنجش سرعت جریان ادرار را دارد، تخلیه میکند. در انتهای تست علاوه بر مقادیر کمی گزارش شده، یک منحنی که نشان دهنده سیر جریان ادرار بوده است، رسم میشود (۱۱).

جریان ادرار به طور کلی میتواند پیوسته یا منقطع باشد. جریان ادرار منقطع وقتی اتفاق می افتد که جریان ادرار بیمار در هر بار مراجعه به سرویس بهداشتی برای تخلیه ادرار، چند بار قطع و وصل شود. جریان ادرار پیوسته نیز میتواند طبیعی و زنگوله ای باشد یا اینکه نوسان دار با چند قله باشد. شکل منحنی جریان ادرار براساس اختلال در انقباض پذیری عضله دترسور مثانه، اختلال انسداد خروجی مثانه یا افزایش فشار شکمی در حین تخلیه ادرار متفاوت باشد (۲۷). منحنی جریان ادرار طبیعی به شکل یک زنگوله پیوسته و بدون شکستگی است، درحالی که در اختلالات تخلیه ادرار انسدادی این منحنی مسطح و با افزایش طول مدت خواهد شد. همچنین در بیماران مبتلا به دیس سینرژی دترسور-اسفنکتر، این الگو به صورت یک منحنی منقطع و ناپیوسته در میاید (۱۱).

تعریف کاربردی: در این مطالعه به منظور ارزیابی فراوانی الگوی طبیعی و غیرطبیعی تخلیه ادرار در بین تمام بیماران، از انجام تست یوروفلومتری توسط پزشک متخصص اورولوژی، قبل و بعد از درمان استفاده خواهد شد.

تغییرات بعد از درمان از نگاه بیمار

تعریف شرحی: یک تفسیر کلی از یک بیماری و شرایط پیچیده که توسط شاخص های عمومی سنجیده میشود. این ابزار های عمومی شدت یک بیماری یا تغییرات کلی وضعیت بیمار بعد از درمان را گزارش می کنند (۳۳).

تعریف کاربردی: در این مطالعه به منظور بررسی تغییرات کلی بیمار بعد از درمان از پرسشنامه PGI-C استفاده خواهد شد.

بررسی متون

کجباف زاده و همکاران در سال ۲۰۱۵ با هدف بررسی تاثیر جریان الکتریکی اینترفرنشیال و یوروتراپی در درمان مثانه کم کار(UAB)در کودکان مبتلا به اختلال تخلیه ادرار، یک مطالعه مداخله ای را در ۳۶ کودک ۱۳-۵ ساله، در قالب دو گروه ۱۸ نفری یوروتراپی استاندارد به تنهایی و یوروتراپی استاندارد همراه با جریان اینترفرنشیال طراحی کردند. کودکانی که مثانه کم کار آنها از طریق یورودینامیک تشیخص داده شده بود و پاسخ مثبتی به درمان دارویی نگرفته بودند، همچنین الگوی جریان ادراری منقطع همراه با سرعت پایین جریان ادرار، طولانی شدن زمان تخلیه ادرار، تخلیه ادرار کمتر از ۳ بار در روز و همراه با زور زدن، PVR بیشتر از ۲۰ میلی لیتر و سن بیش تر از ۵ سال داشتند، وارد این مطالعه شدند. معیار های خروج این مطالعه شامل کودکان مبتلا به عقب ماندگی ذهنی، اختلالات عصبی و مشکلات ساختاری بود. کودکان در هر ۲ گروه ۱۵ جلسه را گذراندند. یوروتراپی استاندارد شامل توضیح و آموزش ساده درمورد عملکرد راه های ادراری و دفعی به کودکان و والدین آنها، مصرف مناسب مایعات، برنامه مناسب تخلیه ادرار بصورت ۲-۳ ساعت یکبار، آموزش شیوه مناسب استفاده از سرویس بهداشتی، حفظ راستای صحیح ستون فقرات حین تخلیه ادرار، حمایت مناسب کف پا در حین تخلیه ادرار و نکات مشابه می شد. جریان اینترفرنشیال نیز بصورت یک جریان ۲۰ دقیقه ای با فرکانس حامل ۴۰۰۰ هرتز با دستگاه الکتروتراپی نوین اعمال شد و الکترود گذاری با استفاده از ۲ جفت الکترود ۲/۵*۳/۵ سانتی متری، به صورت ضربدری روی سمفیزیس پوبیس و ایسکیال توبروزیتی انجام گرفت. شدت جریان تا جایی زیاد شد که یک حس قوی ولی راحت برای کودک ایجاد شود. تاریخچه ادراری و دفعی ۷ روزه، یوروفلومتری، EMG، عفونت مجرای ادراری و الگوی جریان ادراری و اندازه گیری PVR با استفاده از سونوگرافی مثانه از جمله ابزار های برآورد نتیجه در این مطالعه بودند. این مطالعه کاهش معنادار میانگین ظرفیت مثانه را بعد از پیگیری ۱ ساله در گروه یوروتراپی استاندارد همراه با جریان اینترفرنشیال نسبت به گروه یوروتراپی استاندارد به تنهایی نشان داد. همچنین کاهش معنادار میانگین PVR و افزایش میانگین فرکانس مراجعه به سرویس بهداشتی در کودکان گروه یوروتراپی استاندارد همراه با جریان اینترفرنشیال در مقایسه با کودکان گروه یوروتراپی استاندارد به تنهایی را نشان داد. در مقادیر یوروفلومتری، در انتهای دوره درمان و همچنین دوره ی پیگیری ۱ ساله مطالعه، در گروه یوروتراپی استاندارد همراه با جریان اینترفرنشیال نسبت به گروه یوروتراپی استاندارد به تنهایی، سرعت جریان حداکثری ادرار به طور معناداری افزایش و مدت زمان تخلیه ادرار به طور معناداری کاهش یافت. همه ی بیماران در

ابتدا الگوی غیر طبیعی جریان ادرار داشتند. در انتهای دوره پیگیری ۱ ساله ی این مطالعه، الگوی ادرار ۷۷درصد گروه یوروتراپی استاندارد همراه با جریان اینترفرنشیال و ۳۳ درصد گروه یوروتراپی استاندارد به تنهایی طبیعی شد. بنابراین نتایج این مطالعه به نفع جریان اینترفرنشیال برای تخلیه موثر مثانه در کودکان مبتلا به مثانه کم کار با تاثیر روی ویژگی های عصبی و عضلانی هست(۳).

شریفی راد و همکاران در سال ۲۰۱۹ با هدف بررسی تاثیر جریان اینرفرنشیال در بهبود علائم مسیر تحتانی ادراری و مولفه های مرتبط با جریان ادرار در ۲۳ کودک مبتلا به اختلال اولیه گردن مثانه که به لحاظ نورولوژیک سالم بودند، یک مطالعه مداخله ای طراحی کردند. مکانیسم دقیقی که موجب ایجاد این اختلا میشود، انسداد عملکردی خروجی مثانه توضیح داده شده است. کودکان بالای ۴ سال با علائم مجرای ادرای در حداقل ۶ ماه گذشته که به درمان دارویی پاسخ نداده بودند، با شکل منحنی جریان ادرار غیرطبیعی (به صورت مسطح و بدون قله) همراه با تاخیر زمانی بیش از ۶ ثانیه در خاموش شدن فعالیت الکتریکی عضلات کف لگن در EMG این عضلات تا شروع جریان ادرار، سرعت جریان ادرار کم و عدم ثبت فعالیت الکتریکی عضلات کف لگن با EMG در حین تخلیه ادرار وارد این مطالعه شدند. معیارهای خروج مشکلات ساختاری، مثانه عصبی و ثبت فعالیت الکتریکی عضلات کف لگن با EMG در حین تخلیه ادرار بودند. تمام کودکان حاضر در این مطالعه تحت درمان یوروتراپی استاندارد و ۱۵ جلسه جریان اینترفرنشیال به صورت ۲ جلسه در هفته قرار گرفتند. در این مطالعه یوروتراپی استاندارد شامل توضیح و آموزش ساده درمورد عملکرد راه های ادراری و دفعی، مصرف مناسب مایعات، برنامه مناسب تخلیه ادرار بصورت ۲-۳ ساعت یکبار، آموزش شیوه مناسب استفاده از سرویس بهداشتی بود. جریان اینترفرنشیال استفاده شده در این مطالعه با استفاده از دستگاه نوین ۵۱۰A و با فرکانس حامل ۴۰۰۰ هرتز، به مدت ۲۰ دقیقه برای هر کودک اعمال شد. الکترودگذاری بصورت ضربدری با استفاده از ۲ جفت الکترود ۲/۵*۳/۵ سانتی متری روی سمفیزیس پوبیس و ایسکیال توبروزیتی انجام گرفت تا جریان از ناحیه کف لگن و مثانه عبور کند. شدت جریان تا جایی زیاد شد تا کودک یک حس قوی ولی راحت از جریان داشته باشد. یوروفلومتری همراه با EMG ، سونوگرافی مثانه و کلیه، تاریخچه ادراری و دفع، قبل از شروع درمان و ۲ هفته بعد از اتمام درمان مورد بررسی قرار گرفتند. نتیجه این مطالعه نشان داد به دنبال استفاده از جریان اینترفرنشیال، میانگین سرعت جریان متوسط و حداکثری ادرار به طور معنی داری افزایش و میانگین زمان خاموش شدن فعالیت الکتریکی عضلات کف لگن در EMG تا شروع جریان ادرار و میانگین میزان حجم باقی مانده ادرار بعد از تخلیه ((PVRبه طور معنی داری کاهش یافت(۱).

لادی سیدیان و همکاران در سال ۲۰۲۰ با هدف بررسی تاثیر استفاده از جریان اینترفرنشیال به صورت درمان کمکی تمرینات عضلات کف لگن در ۳۴ کودک مبتلا به اختلالات مثانه و روده ای غیرعصبی ((BBD، یک مطالعه مداخله ای طراحی کردند. کودکان بالای ۵ سال با سابقه علائم بالینی مجاری تحتانی ادراری، که معیار های) Rome IVپرسشنامه معتبر برای تشخیص یبوست عملکردی) و همچنین الگوی غیر طبیعی منحنی جریان ادرار (الگویی غیر از شکل زنگوله ای) را نیز داشتند، وارد مطالعه شدند. معیارهای خروج مطالعه شامل اختلالات ساختاری، ناهنجاری های نخاعی، مثانه یا روده عصبی و تاخیر رشدی بودند. شرکت کنندگان در این مطالعه طیفی از علائم متفاوت مجاری ادراری شامل عفونت های عود کننده ادراری، بی اختیاری ادراری، احساس اضطرار برای ادرار و نگه داشتن ادرار و همچنین یبوست را برای مدت حداقل ۳ ماه بدون پاسخ به درمان های روتین پزشکی داشتند. در این مطالعه، کودکان در ۲ گروه جریان اینترفرنشیال همراه با تمرینات عضلات کف لگن و تمرینات عضلات کف لگن به تنهایی قرار گرفتند. در مان در هر ۲ گروه بصورت ۱۰ جلسه انجام شد. جریان اینترفرنشیال توسط یک فیزیوتراپیست به مدت ۲۰ دقیقه، با دستگاه نوینDS ۱۲۶ دوکاناله، با فرکانس حامل ۴۰۰۰ هرتز استفاده شد. الکترود گذاری با استفاده از ۲ جفت الکترود ۲/۵*۳/۵ سانتی متری و به صورت ضربدری در ناحیه سمفیزیس پوبیس و ایسکیال توبروزیتی انجام گرفت. شدت جریان تا جایی زیاد شد تا کودک یک حس قوی ولی راحت از جریان داشته باشد. مطابق با گزارش انجمن بین المللی دفع کودکان، کاهش ۹۹-۵۰ درصدی و ۱۰۰ درصدی در شاخص های Rome IV، تاریخچه ادراری و دفع و پرسشنامه مجاری تحتانی ادراری، به ترتیب به عنوان "پاسخ نسبی" و "پاسخ کامل" به درمان در کودکان مبتلا به اختلالات مثانه و روده ای تلقی می شوند. بر این اساس نتایج

این مطالعه نشان می دهد که ۶۴/۷ درصد گروه جریان اینترفرنشیال همراه با تمرینات عضلات کف لگن در مقایسه با ۲۹/۴ درصد گروه تمرینات عضلات کف لگن، "پاسخ کامل" به درمان داده اند. همه ی کودکان شرکت کننده در این مطالعه در ابتدا الگوی جریان ادرار غیر طبیعی داشتند، اما در انتهای درمان، تفاوت معناداری به لحاظ معیار های یوروفلومتری بین ۲ گروه مشاهده نشد. میانگین فرکانس اپیزود های بی اختیاری ادراری روزانه، در همه کودکان گروه جریان اینترفرنشیال همراه با تمرینات عضلات کف لگن در مقایسه با ۲۵ درصد گروه تمرینات عضلات کف لگن به تنهایی، بعد از درمان و بعد از دوره ی پیگیری ۶ ماهه، به طور معناداری بهبود یافت.(۲۳)

Vittin و همکاران در سال ۲۰۲۲ با هدف بررسی تاثیر ۸ هفته جریان اینترفرنشیال در درمان بزرگسالان مبتلا به یبوست مزمن یک کارآزمایی بالینی تصادفی دو سو کور را طراحی کردند. بیماران بالای ۱۸ سال و از هر دو جنس وارد این مطالعه شدند. از جمله معیارهای خروج مرتبط این مطالعه می توان به بارداری، شیردهی، اختلال کف لگن که نیازمند جراحی باشد مثل پرولاپس درجه بالای رکتوم، دستگاه الکتریکی کارگذاری شده در بدن مثل ضربان ساز قلبی یا تحریک کننده ی نخاعی اشاره کرد. جریان اینترفرنشیال با فرکانس حامل ۴۰۰۰ هرتز و فرکانس ضربان ۱۶۰-۸۰ هرتز و شدت زیر ۳۳ میلی آمپر، با استفاده از دو الکترود در سطح شکمی (زیر حاشیه دنده ای) و دو الکترود دیگر دو طرف ستون فقرات (بین T۹ و L۲) ،با هدف عبور جربان سینوسی از عرض بدن، اعمال شد. در گروه کنترل تمام نکات آموزش داده شده برای استفاده خانگی دستگاه مشابه بود با این تفاوت که دستگاه هیچ جریانی تولید نمیکرد. به علاوه بیماران و درمانگران دخیل در این پژوهش نسبت به گروه بندی ها کور بودند. درمان در هر ۲ گروه بصورت ۱ ساعت و روزانه، به مدت ۸ هفته انجام گرفت. نتیجه این مطالعه نشان داد نمره ارزیابی بیمار از نمرات پرسشنامه علائم یبوست (PAC-SYMدر گروه درمان نسبت به گروه کنترل بصورت معناداری کاهش پیدا کرد. به علاوه نمره ارزیابی بیمار از نمرات کیفیت زندگی مرتبط با یبوست (PAC-QOLدر گروه درمان نسبت به گروه کنترل به طور معناداری افزایش یافت. اما حرکات کامل خودبه خودی روده، میانگین زمان حرکات انتقالی روده، اتساع پذیری روده، مانومتری روده در دو گروه تفاوت معناداری باهم نداشتند(۲۱).

Moore و همکاران در سال ۲۰۱۹ به منظور بررسی تاثیر کوتاه مدت و بلند مدت جریان اینترفرنشیال در ۳۳ زن مبتلا به یبوست عملکردی یک کارآزمایی بالینی تصادفی یک سو کور (بیماران نسبت به گروه بندی ها کور بودند) را طراحی کردند. از جمله معیار ورود در این مطالعه میتوان به زنان بالغ ۷۵-۱۸ ساله اشاره کرد. معیارهای خروج مهم این مطالعه شامل بارداری، یبوست به دنبال مصرف دارو/ بیماری متابولیک/ بیماری سیستمیک یا بیماری عصبی، سابقه بیماری مزمن و جدی در فرد، سابقه جراحی در روده یا رکتوم و سابقه آسیب یا جراحی که اعصاب ساکرال را تحت تاثیر قرار داده باشد، بود. جریان اینترفرنشیال با فرکانس حامل ۴۰۰۰ هرتز و فرکانس ضربان ۱۶۰-۸۰ هرتز با استفاده از دو الکترود سطحی به کار برده شد. در گروه درمان الکترد ها بصورت ضربدری در سطح شکمی و پشتی به کار برده شدند به این صورت که یک الکترود یک کانال را در سطح شکمی راست و الکترود دیگر همان کانال را در سطح پشتی چپ قرار دادند تا جریان از شکم عبور کند. در گروه کنترل همه چیز به جز الکترود گذاری شبیه به گروه درمان بود، به این صورت که الکترود ها بصورت جانبی (برای مثال سطح پشتی راست و سطح شکمی راست برای الکتروهای یک کانال) قرار داده شدند با این فرض که اینگونه جریان بصورت زیر جلدی عبور می کند. در این مطالعه استفاده از جریان جهت کاربرد توسط بیماران بصورت یک ساعت روزانه به مدت ۶ هفته آموزش داده شد. نتیجه این مطالعه نشان داد ۵۳ درصد گروه درمان در مقایسه با ۱۲/۵ درصد گروه کنترل توانستند در هفته ۸-۶ درمان حداقل ۳ بار دفع خود به خودی و بدون استفاده از ملین (پیامد اولیه این مطالعه) داشته باشند. هرچند در بین پیامد های ثانویه، تفاوت معنادار آماری در گروه درمان نسبت به گروه کنترل در پیامد VAS برای علائم و کاهش ۵۰ درصدی استفاده از داروهای ملین مشاهده شد، تفاوت های چشم گیر در تغییر دو پیامد PAC-SYM و حرکات کامل خودبه خودی روده در طی یک هفته دیده شد. در کل نتایج این مطالعه شواهد خوبی را برای پشتیبانی از استفاده از جریان اینترفرنشیال شکمی در بهبود علائم روده ای در بیماران مبتلا به یبوست مزمن فراهم نمود(۲۴).

دو مطالعه اخیر که درمورد کاربرد جریان اینترفرنشیال در اختلال یبوست در بزرگسالان انجام شده است، هرچند جدید هستند و

این موضوع نیز در جمعیت بزرگسالان کمتر مورد توجه قرار گرفته است، در سیستماتیک ریویو های مرتبط به لحاظ روش کار مورد انتقاد قرار گرفته اند(۲۵).

طرح محصول پایان نامه است؟

پایان نامه نیست

جدول زمان بندی اجرای طرح

ردیف	مراحل اجرایی	زمان کل										
۱	نگارش پروپوزال	۱ ماه	۱۰	۹	۸	۷	۶	۵	۴	۳	۲	۱
۲	تصویب طرح و گرفتن کد اخلاق	۱ ماه	۱۰	۹	۸	۷	۶	۵	۴	۳	۲	۱
۳	اجرای طرح و جمع آوری داده ها	۶ ماه	۱۰	۹	۸	۷	۶	۵	۴	۳	۲	۱
۴	تحلیل داده ها	۱ ماه	۱۰	۹	۸	۷	۶	۵	۴	۳	۲	۱
۵	گزارش نتایج و انتشار مقاله	۱ ماه	۱۰	۹	۸	۷	۶	۵	۴	۳	۲	۱

کل مدت زمان اجرا : ۱۰ ماه

روش اجرا

روش اجرا

پس از تصویب پروژه، گرفتن کد اخلاق نمونه گیری را با روش غیراحتمالی دردسترس انجام خواهد شد. به این صورت که پزشک متخصص اورولوژی بیماران مبتلا به اختلال تخلیه ادرار مراجعه کننده به درمانگاه را معاینه و ارزیابی خواهند کرد. معاینات پزشک متخصص شامل گرفتن تاریخچه و پرسش درمورد علائم بالینی فرد، ارزیابی کامل بیمار به لحاظ درگیری های نورولوژی، انواع اختلالات اورولوژی، سیستومتری (به منظور سنجش فشار دترسور در فاز پر شدن و فشار جریان ادرار در فاز تخلیه مثانه(۱۰)) و تست های یورودینامیک و سونوگرافی با مثانه پر و خالی، به منظور افتراق نوع اختلال تخلیه ادراری خواهد بود. ارزیابی نورولوژی شامل بررسی تون ناحیه آنال، کنترل ارادی دریچه آنال، بررسی رفلکس های ناحیه تناسلی و رفلکس های اندام تحتانی و بررسی حس ناحیه تناسلی می شود. سپس درصورتی که بیمار مراجعه کننده، با معیار های ورود مطالعه پیش رو همخوانی داشته باشد، مطابق با تشخیص پزشک متخصص وارد این طرح خواهند شد. همه بیماران درمان پایه روتین مرتبط با اختلال تخلیه ادرار مثل درمان دارویی را دریافت خواهند کرد. سپس به همه بیماران تاریخچه ادراری ۳ روزه را داده و طریقه تکمیل آن را آموزش داده

خواهد شد. پزشک متخصص از بیمار می خواهند که از روز بعد ویزیت به مدت ۳ روز این برگه را به دقت تکمیل کرده و مقادیر مایعات مصرفی و نوع مایعات مصرفی و دفعات مراجعه به سرویس بهداشتی را به صورت کامل در طی ۳ روز ثبت کنند. سپس به بیمار مقدمه ای از درمان پیش رو توضیح داده خواهد شد و ایشان را به همراه پرونده کامل مدارک پزشکی که شامل تست یورودینامیک و سونوگرافی می باشد، برای ۳ روز آینده به کلینیک های توانبخشی دانشگاه علوم پزشکی ایران ارجاع خواهند داد. پزشک متخصص اورولوژی همکار در طرح پیش رو به عنوان ارزیابی کننده پیامد های یورودینامیک نسبت به گروه بندی بیماران که در ادامه انجام خواهد گرفت، بی اطلاع خواهد ماند.

در کلینیک فیزیوتراپی، در ابتدا به تمام افراد توضیح جامعی از روند کامل کار و هدف از انجام تحقیق داده خواهد شد و سپس از همه آن ها رضایت نامه شخصی اخذ خواهد شد. به بیماران توضیح داده خواهد شد که ۲ گروه درمانی وجود دارد که همه درمان ها در هر ۲ گروه به جز نحوه اعمال جریان الکتریکی اینترفرنشیال مشابه هستند و گروه بندی به صورت کاملا تصادفی صورت خواهد گرفت و امکان قرار گرفتن آنها در هر کدام از گروه ها با احتمال یکسان وجود دارد. همچنین به آنها این اطمینان داده خواهد شد که اگر در انتهای پروژه تغییر معناداری در پیامدهای تحقیق دیده شد، درمان انتخابی برای گروهی که آنرا دریافت نکردند به صورت کامل انجام خواهد گرفت.

تاریخچه ادراری ۳ روزه تکمیل شده را از بیمار تحویل گرفته خواهد شد و از بین اطلاعات موجود در آن، فرکانس مراجعه به سرویس بهداشتی در ۲۴ ساعت استخراج خواهد شد. سپس با استفاده از لیست کامپیوتری شماره های تصادفی، بیماران به ۲ گروه تصادفی درمان و کنترل، با نسبت ۱:۱ با بلوک های ۴ تایی تقسیم خواهند شد. در ادامه پرسشنامه خودساخته جهت جمع آوری اطلاعات فردی هر بیمار شامل سن، قد و وزن در ابتدای جلسه ای اول هر ۲ گروه تکمیل خواهد شد. پس از آن به همه بیماران نسخه فارسی پرسشنامه ICIQ-FLUTS و PFDI-۲۰ داده خواهد شد تا بیماران با توجه به آن به ترتیب، به شدت علائم ادراری تحتانی و شدت علائم کف لگن خود نمره بدهند.

در جلسه آخر درمان، مجددا نسخه فارسی پرسشنامه ICIQ-FLUTS به بیماران داده میشود تا نمره دهی آنها به شدت علائم ادراری تحتانی با قبل از شروع درمان مورد مقایسه قرار بگیرد. همچنین در این جلسه به بیماران نسخه فارسی پرسشنامه تغییرات عمومی بیمار (PGI-C) داده میشود تا از دید خودشان به تغییرات کلی وضعیت خود بعد از درمان نمره دهی کنند. درنهایت، از بیمار خواسته میشود تا تاریخچه ادراری ۳ روزه را دوباره تکمیل کرده و بعد از گذشت ۲ هفته جهت انجام مجدد تست یوروفلومتری و معاینه و ارزیابی علائم بالینی همراه با برگه تکمیل شده تاریخچه ادراری ۳ روزه به پزشک متخصص اورولوژی مراجعه کند. همچنین یک دوره پیگیری ۳ ماهه در این مطالعه در نظر گرفته شده است، که بعد از گذشت این دوره یک بار دیگر نیز انجام تست یوروفلومتری تکرار خواهد شد و علائم بالینی بیماران بررسی میشود تا تاثیر طولانی مدت یوروتراپی روتین به تنهایی و یوروتراپی روتین به همراه جریان اینترفرنشیال در علائم بالینی و یافته های یوروفلومتری بیماران مبتلا به اختلال تخلیه ادرار انسدادی عملکردی نیز مورد بررسی قرار بگیرد.

پروتکل درمانی

۱) درمان پایه مشابه در هر ۲ گروه درمان و کنترل: در هر دو گروه یوروتراپی روتین و تمرین درمانی مرتبط با اختلال تخلیه ادرار، به عنوان درمان های پایه انجام خواهد گرفت. برنامه یوروتراپی روتین به این صورت است که درمورد عملکرد راه های ادراری و دفعی، مصرف مناسب مایعات، برنامه مناسب تخلیه ادرار بصورت ۲-۳ ساعت یکبار، آموزش شیوه مناسب استفاده از سرویس بهداشتی و درصورت نیاز شیوه استفاده درست از سوند یکبارمصرف (سوند فولی)، توسط فیزیوتراپیست متخصص اختلالات کف لگن آموزش داده خواهد شد(۱). در آموزش شیوه درست استفاده از سرویس بهداشتی، راستای صحیح ستون فقرات حین تخلیه ادرار اهمیت دارد. همچنین به بیماران آموزش داده می شود که در حین تخلیه ادرار به خوبی ران ها را از هم دور، عضلات شکم و کف لگن را ریلکس کنند و جریان ادرار را در طول زمان تخلیه مثانه قوی نگه دارند. به همه بیماران توصیه میشود که مانند دفع ادرار، برنامه منظمی هم برای دفع مدفوع داشته باشند و از غذاهای با فیبر زیاد استفاده کنند.(۳) درمان یوروتراپی روتین به مدت

۱۰ جلسه ۲۰ دقیقه ای به صورت هفته ای ۲ جلسه در کلینیک فیزیوتراپی سرپایی دانشکده توانبخشی (یا به صورت جلسات برخط) انجام خواهد گرفت. تمرین درمانی مرتبط با اختلال تخلیه ادرار شامل تنفس دیافراگماتیک، تمرینات ریلکسیشن عضلات کف لگن، انقباضات عضلات کف لگن و ایجاد ریلکسیشن ۳۰ ثانیه عضلات کف لگن به دنبال ۱۰ ثانیه انقباض این عضلات میشود (۳, ۱۸, ۴۳).

۲) جریان الکتریکی اینترفرنشیال در گروه درمان: در بیماران گروه درمان، از جریان اینترفرنشیال با فرکانس حامل ۴۰۰۰ هرتز و فرکانس ضربان ۱۶۰-۸۰ هرتز با استفاده از دستگاه نوین (۲ کاناله/ ساخت ایران، اصفهان) به مدت ۳۰ دقیقه استفاده خواهد شد. از ۴ الکترود خودچسبان ۵*۵ سانتی متر برای اعمال جریان روی سطح پوست استفاده خواهد شد. الکترود گذاری به صورت ضربدری انجام خواهد گرفت، به این صورت که یک الکترود از هر کانال روی سمفیزیس های پوبیس و الکترود های دیگر بصورت متقابل روی توبروزیتی های ایسکیال قرار میگیرند تا جریان الکتریکی از ناحیه کف لگن عبور کند(۱, ۱۸). شدت جریان تا جایی زیاد میشود که یک حس قوی از جریان توسط بیماران گزارش شود. درصورتی که حس درد و ناخوشایندی توسط بیماران گزارش شود، جریان تا سطح قابل قبول کم خواهد شد. جریان اینترفرنشیال به مدت ۱۰ جلسه ۳۰ دقیقه ای به همراه ۲۰ دقیقه یوروتراپی روتین در هرجلسه به صورت هفته ای ۲ جلسه ، در کلینیک فیزیوتراپی دانشکده توانبخشی انجام خواهد گرفت.

۳) جریان الکتریکی اینترفرنشیال در گروه کنترل: در گروه کنترل نیز جریان الکتریکی اینترفرنشیال با دستگاه، تنظیمات و روش الکترودگذاری مشابه با گروه درمان استفاده خواهد شد. با این تفاوت که شدت خروجی دستگاه در حد احساس اولیه بیمار تنظیم می شود و سپس بعد از گذشت ۱ دقیقه خروجی دستگاه قطع خواهد شد (۳۱, ۴۴).

مشخصات ابزار جمع آوری اطلاعات و نحوه جمع آوری

۱) پرسشنامه خودساخته جهت جمع آوری اطلاعات فردی

یک پرسشنامه که توسط محققین این مطالعه ساخته شده است و به وسیله آن خصوصیات فردی بیماران از جمله سن، قد، وزن، شاخص توده بدنی(BMI)گردآوری خواهد شد.

۲) تست یوروفلومتری

مطالعات یورودینامیک تنها معیار استاندارد برای تشخیص اختلال تخلیه ادرار است. این تست یک مطالعه عملکردی از مسیر ادراری تحتانی است که توالی انجام بالینی آن به این صورت است که ابتدا یوروفلومتری ساده و بدون کاربرد سوند انجام میشود و بعد از آن حجم باقی مانده ادرار بعد از تخلیه با انجام سونوگرافی با مثانه پر و خالی اندازه گیری خواهد شد (۱۰). یوروفلومتری یک تست یورودینامیک غیرتهاجمی است که تخلیه ادرار را قبل درمان و در دوره پیگیری مداخلات درمانی مورد ارزیابی قرار میدهد. در این تست بیماران تخلیه ادرار خود را در مخزنی که قابلیت اندازه گیری سرعت تخلیه ادرار را دارد، انجام میدهند. در انتهای این تست یک منحنی جریان ادرار رسم میشود و متغیرهای مربوطه به صورت اعداد کمی گزارش میشوند (۱۱, ۳۴). یوروفلومتری در بیماران در حالتی که مثانه آنها پر است در یک محیط خصوصی با استفاده از تکنیک استاندارد اجرا می شود. با استفاده از این تست ۳ متغیر الگوهای جریان ادرار، سرعت حداکثری جریان ادراری و مدت زمان دفع ادرار بدست می آید. الگوهای غیرطبیعی جریان ادرار شامل الگوهای منقطع، دندانه اره ای و الگوهای با زمان جریان ادرار طولانی می باشند. کاهش سرعت حداکثری جریان ادرار به کمتر از ۱۵ میلی لیتر بر ثانیه، افزایش زمان تا رسیدن این سرعت به بیشتر از ۵ ثانیه و افزایش مقدار حجم باقی مانده ادرار به بیشتر از ۵۰ میلی لیتر میتواند نشانه اختلال تخلیه ادرار باشد (۱۳, ۳۵). برای افتراق بین اختلال تخلیه ادراری انسدادی عملکردی و اختلال تخلیه ادرار به دنبال اختلال عملکرد دترسور، نیاز به انجام تست یورودینامیک تهاجمی می باشد که در آن فشار دترسور نیز اندازه گیری شود. مهم ترین مولفه به نفع تشخیص اختلال تخلیه ادراری انسدادی، شاخص انسداد مجرای مثانه است که اگر بیش تر از ۱۸ باشد این اختلال تشخیص داده میشود. این شاخص تشخیصی از طریق حاصل تفریق ۲/۲ برابر جریان

حداکثر ادرار از فشار دترسور در قله جریان ادرار بدست می آید و مطالعات حساسیت ۹۰ درصدی این شاخص را برای تشخیص اختلال تخلیه ادرار انسدادی نشان داده اند (۵، ۱۲). در تست یورودینامیک، جریان حداکثر ادرار مساوی و کمتر از ۱۵ میلی لیتر بر ثانیه، فشار دترسور در جریان حداکثر ادرار مساوی و کمتر از ۲۰ و مقدار PVR بیشتر از ۱۰ درصد به نفع تشخیص اختلال تخلیه ادرار به دنبال اختلال عملکردی دترسور مثانه می باشد (۱۲).

۴) تاریخچه ادراری ۳ روزه (۳۶)

جدولی که درآن دفعات تخلیه ادرار و حجم ادرار تخلیه شده در هر بار، فرکانس اپیزود های بی اختیاری ادراری، استفاده از پد (در موارد بی اختیاری) و استفاده از سوند یکبار مصرف(در موارد اختلالات احتباسی)، اطلاعات مربوط به مصرف مایعات از جمله مقادیر و نوع مایعات مصرفی در طی زمان های معین توسط بیمار ثبت می شود. در موارد اختلالات احتباسی توصیه شده است از تاریخچه های ادراری ۳ روزه یا بیشتر از آن استفاده شود.(۹، ۳۷) تاریخچه های ادراری جزئی از پروتکل یورودینامیک انجمن بین المللی بی اختیاری هستند و اطلاعات مفیدی درمورد مثانه در فاز پر شدن مثل ظرفیت حداکثر و عملکردی مثانه فراهم میکند (۳۶).

در این مطالعه از تاریخچه ادراری ICIQ استفاده خواهد شد که در آن بیمار فرکانس ادرار، مقدار ادرار تخلیه شده در هربار و مقادیر مربوط به مایعات مورد استفاده (زمان استفاده، مقدار استفاده، نوع نوشیدنی) در ۳ روز را ثبت میکند. همچنین در این تاریخچه ستون اضافی برای ثبت مربوط به احساس پر شدن مثانه و استفاده از پد نیز وجود دارد. این تاریخچه به شدت از سمت انجمن بین المللی بی اختیاری توصیه شده است (۳۸).

۵) پرسشنامه PFDI-۲۰ (۳۹)

پرسشنامه PFDI-۲۰ از سمت انجمن بین المللی بی اختیاری به عنوان ابزاری برای ارزیابی اختلالات کف لگن، به شدت توصیه شده است (۳۹). این پرسشنامه شامل ۲۰ سوال در ۳ شاخص متفاوت میشود که شدت علائم اختلالات کف لگن را در ۳ شاخص ذکرشده می سنجد. هر سوال در این پرسشنامه براساس علائم بیماران میتواند در ۴ سطح پاسخ داده شود. جمع نمرات در هر شاخص ۱۰۰ و در کل پرسشنامه ۳۰۰ است. در این پرسشنامه هرچه نمره ی فرد بالاتر باشد، شدت اختلال او بیشتر است. ۳ شاخص این پرسشنامه به شرح زیر است:

۱) علائم پرولاپس ناحیه تناسلی(POPDI):شامل ۶ سوال

۲) علائم روده ای-مقعدی(CRADI):شامل ۸ سوال

۳) علائم ادراری(UDI):شامل ۶ سوال، که سوال ۱۶ و ۱۷ به ترتیب مخصوص بی اختیاری ادراری اورژانسی و استرسی هستند (۴۰).

۶) نسخه فارسی پرسشنامه (ICIQ-FLUTS (۴۱

پرسشنامه ICIQ-FLUTS یک پرسشنامه خوداظهاری مورد استفاده در بالین و تحقیقات است که علائم ادراری تحتانی و تاثیر آنها بر کیفیت زنان را مورد بررسی قرار میدهد. این پرسشنامه شامل ۱۲ سوال میشود و همه سوالات شامل ۲ قسمت میشوند؛ قسمت اول مربوط به نمره دهی به شدت علائم ادراری با یک شاخص لیکرت است که فرد میتواند به شدت هر علامت از ۰ تا ۴ نمره دهی کند. هر چه نمره بالاتر باشد به این معناست که شدت علامت نیز بیشتر است. قسمت دوم درمورد شدت اذیت و آسیبی است که هر علامت بالینی برای بیمار ایجاد میکند. در قسمت دوم نیز فرد میتواند از ۰ تا ۱۰ نمره دهی کند و هرچقدر این نمره بیشتر باشد، به این معناست که آن علامت بیشتر برای فرد اذیت کننده است. سوالات این پرسشنامه در ۳ دسته تقسیم بندی می شوند:

۱- علائم فاز پرشدن مثانه: شامل ۴ سوال

۲- علائم فاز تخلیه مثانه: شامل ۳ سوال

۳- علائم بی اختیاری: شامل ۵ سوال (۴۱)

۷) پرسشنامه GRCS

این پرسشنامه ها برای کمی سازی تغییرات بیماران در طی زمان کاربرد دارد و معمولا به دنبال کاربرد یک مداخله درمانی و به منظور بررسی اثر گذاری یک مداخله خاص درمانی یا تغییرات یک بیماری در طی زمان استفاده می شود. این دسته از پرسشنامه ها با پرسش یک سوال از بیمار می خواهند تغییرات خود در طی زمان و عموما به دنبال یک درمان خاص را نمره دهی کنند. در این مقیاس "۰" به معنای "هیچ تغییری نکردم" است و نمرات منفی به معنای "بدتر شدم" و نمرات مثبت به معنای "بهتر شدم" می باشد. این پرسشنامه ها با نام های مختلفی برای استفاده در دسترس هستند (۴۲).
در این مطالعه ما از پرسشنامه Patient Global Impression of Change استفاده خواهیم کرد. این پرسشنامه شامل یک سوال با هفت پاسخ قابل انتخاب است که تغییرات فرد را به دنبال یک مداخله خاص ارزیابی می کند. پاسخ های این سوال شامل خیلی خیلی بدتر شدم، خیلی بدتر شدم، بدتر شدم، تغییری نکردم، بهتر شدم، خیلی بهتر شدم، خیلی خیلی بهتر شدم می شود(۳۳).

روش محاسبه حجم و تعداد نمونه

در مطالعه کجباف زاده و همکاران در سال ۲۰۱۵، میانگین (انحراف معیار) جریان حداکثر ادرار (Qmax)در گروه مداخله و گروه کنترل بعد از دوره پیگیری ۱ ساله به ترتیب ۲۱ (۸/۳) و ۱۲/۸ (۴/۸) گزارش شده است. بر این اساس، حجم نمونه در سطح اطمینان ۹۵ ٪ و توان آماری ۸۰ ٪ با استفاده از نرم افزار STATA محاسبه و با در نظر گرفتن ۱۰ درصد ریزش در نمونه ها تعداد ۱۴ نمونه در هر گروه محاسبه گردید (۳).

محدودیتهای اجرایی طرح و روش کاهش آنها

این مطالعه به علت دوره پیگیری، ممکن است با ریزش بیماران روبه رو شود. به منظور کاهش این مسئله تلاش شده است که تا جای ممکن دوره پیگیری را کوتاه انتخاب شود و به علاوه اهمیت مراجعه مجدد به پزشک متخصص اورولوژی بعد از دوره پیگیری به خوبی برای بیماران روشن گردد.

آیا پژوهش دارای آزمایش تخصصی و یا تکنیک اختصاصی می باشد؟

بلی

ترجمان دانش

مخاطبین طرح

ارائه دهندگان خدمت(پزشک، پرستار، ماما و ....)

اگر مخاطب مسئولین و مدیران هستند توضیحات لازم را قید بفرمائید

اگر مخاطب ارائه دهندگان خدمت هستند توضیحات لازم را قید بفرمائید

در صورتی که درمان با جریان اینترفرشیال در زنان مبتلا به اختلال تخلیه ادرار، بتواند بهبود معناداری در علائم کلینیکی و یافته های یوروفلومتری این افراد ایجاد کند، در آینده درمانگران حوزه اختلالات کف لگن (فیزیوتراپیست ها- اورولوژیست ها، فلوشیب های اختلالات کف لگن، گایناکولوژیست ها) میتوانند از این درمان برای بیماران مبتلا به اختلال ادراری استفاده کنند.

اگر مخاطب عامه مردم و بیماران هستند توضیحات لازم را قید بفرمائید

اگر مخاطب شرکت ها و صنایع هستند توضیحات لازم را قید بفرمائید

اگر مخاطب سایر نهادها هستند توضیحات لازم را قید بفرمائید

روش به کارگیری، اطلاع رسانی و تبادل دانش

ارائه در کنفرانس ها و سمینارهای خارجی، ارائه در کنفرانس ها و سمینارهای داخلی، ارسال خلاصه یا گزارش کامل طرح یا مقاله حاصل از آن برای استفاده کنندگان بالقوه آن ، انتشار مقاله در مجله های علمی- پژوهشی خارجی، انتشار مقاله در مجله های علمی- پژوهشی داخلی ، قرار دادن متن کامل گزارش یا خلاصه ای از آن در وب سایت به منظور دسترسی استفاده کنندگان بالقوه به آن

توضیح روش های مورد نظر شما برای به کارگیری نتایج

تمامی گروه های درمانی در حوزه اختلالات کف لگن اعم از فیزیوتراپیست ها، پزشکان متخصص اورولوژی، متخصصین زنان و زایمان میتواند از نتایج طرح پیش رو در درمان بیماران مبتلا به اختلال تخلیه ادراری بهره مند گردند. فیزیوتراپیست ها با ارجاع این بیماران از سوی پزشکان می توانند از روش ها و متدهای ذکر شده برای درمان این بیماران استفاده کرئه و متد درمانی موثر را به پروتکل درمانی خود بیافزایند. مجری طرح به عنوان فیزیوتراپیست متخصص حوزه اختلالات کف لگن پس از انتشار نتایج و مشخص شدن قدرت تاثیرگذاری درمان پیش رو می تواند با استفاده از تحریک الکتریکی سطحی در نواحی ذکر شده منجر به بهبود اختلال تخلیه ادراری در افراد مبتلا گردد.

ذینفع اول (فرد/سازمان)

بیماران مبتلا به اختلال تخلیه ادراری

نمره اهمیت ذینفع اول (از یک تا پنج)

پنج

نمره قدرت ذینفع اول (از یک تا پنج)

دو

ذینفع دوم (فرد/سازمان)

گروه فیزیوتراپی دانشکده توانبخشی دانشگاه علوم پزشکی ایران

نمره اهمیت ذینفع دوم (از یک تا پنج)

سه

نمره قدرت ذینفع دوم (از یک تا پنج)

دو

ذینفع سوم (فرد/سازمان)

درمانگران و فیزیوترایست ها حوزه اختلالات کف لگن

نمره اهمیت ذینفع سوم (از یک تا پنج)

پنج

نمره قدرت ذینفع سوم (از یک تا پنج)

یک

ذینفع چهارم (فرد/سازمان)

متخصصین اورولوژِی_ زنان و زایمان و فلوشیپ های کف لگن

نمره اهمیت ذینفع چهارم (از یک تا پنج)

چهار

نمره قدرت ذینفع چهارم (از یک تا پنج)

سه

متغیرهای طرح

عنوان متغیر	نقش متغیر	نوع متغیر	تعریف علمی	نحوه اندازه گیری	مقیاس
حجم باقی مانده ادرار بعد از تخلیه	وابسته Dependent	کمی / پیوسته quantitative/continuous	به باقی مانده ادرار در مثانه بعد از تخلیه ادرار اطلاق میشود. برای تعیین این میزان از سوند پس از اتمام تخلیه ادرار استفاده می شود (۵, ۱۱). اگر مقدار PVRبیش تر از ۱۰۰ میلی لیتر باشد، به نفع تشخیص اختلال احتباس ادراری است) ۲۰ .(	سونوگرافی	میلی لیتر
جریان حداکثر ادرار (Qmax)	وابسته Dependent	کمی / پیوسته quantitative/continuous	جریان حداکثر ادرار که با علامت Qmax در مطالعات نشان داده میشود به معنای بیشترین سرعت جریان ادرار اندازه گیری شده است و با واحد میلی لیتر بر ثانیه گزارش میشود (۱۰). به طور کلی در اختلالات تخلیه ادرار جریان حداکثر ادرار کاهش می یابد. مطالعات نشان داده اند که مقادیر کمتر از ۱۵ میلی لیتر بر ثانیه در زنان به نفع تشخیص اختلال تخلیه ادرار است) ۱۳.(	یوروفلومتری	میلی لیتر بر ثانیه
مدت زمان تخلیه ادرار	وابسته Dependent	کمی / پیوسته quantitative/continuous	به طول مدت زمان کلی تخلیه ادرار که میتواند شامل وقفه های متعدد نیز بشود، زمان تخلیه	یوروفلومتری	ثانیه

مقیاس	نحوه اندازه گیری	تعریف علمی	نوع متغیر	نقش متغیر	عنوان متغیر
		ادرار گفته میشود. در صورتی که منحنی تخلیه ادرار پیوسته و بدون وقفه باشد زمان تخلیه ادرار و زمان جریان ادرار یکسان خواهند بود) ۱۰ .(			
-	سونوگرافی	کارایی تخلیه مثانه BVE)) یک متغیر برای سنجش انقباض پذیری مثانه در برابر مقاومت مجرای ادراری می باشد. این متغیر مقدار حجم تخلیه شده مثانه را به صورت درصدی از ظرفیت کلی مثانه بیان می کند (۲۲ .(	کمی / پیوسته quantitative/continuous	وابسته Dependent	کارایی تخلیه مثانه
-	یوروفلومتری	در تست یوروفلومتری فرد ادرار خود را دریک مخزن که قابلیت سنجش سرعت جریان ادرار را دارد، تخلیه میکند. در انتهای تست علاوه بر مقادیر کمی گزارش شده، یک منحنی که نشان دهنده سیر جریان ادرار بوده است، رسم میشود (۱۱). منحنی جریان ادرار طبیعی به شکل یک زنگوله پیوسته و بدون شکستگی است، درحالی که در اختلالات تخلیه ادرار انسدادی این منحنی مسطح و با افزایش طول مدت خواهد شد. همچنین در بیماران مبتلا به دیس سینرژی دترسور-اسفنکتر، این الگو به صورت یک منحنی منقطع و ناپیوسته در می آید).۱۱)	کیفی / اسمی / Qualitative nominal	وابسته Dependent	الگوی طبیعی تخلیه ادرار
-	تاریخچه ادراری ۳ روزه	؟	کمی / گسسته quantitative/ Discrete	وابسته Dependent	فرکانس استفاده از سرویس بهداشتی
-	نسخه فارسی پرسشنامه ICIQ-FLUTS	نشانه های ذهنی یک بیماری که توسط بیمار، مراقب یا خانواده اش درک میشود و را به سمت پیدا کردن درمان سوق میدهد. این دسته از نشانه ها معمولا در شرح حال گیری از بیمار به دست می آیند و معمولا کیفی هستند).۱(	کمی / پیوسته quantitative/continuous	وابسته Dependent	شدت علائم ادراری تحتانی

مقیاس	نحوه اندازه گیری	تعریف علمی	نوع متغیر	نقش متغیر	عنوان متغیر
-	نسخه فارسی پرسشنامه PGI-C	یک تفسیر کلی از یک بیماری و شرایط پیچیده که توسط شاخص های عمومی سنجیده میشود. این ابزار های عمومی شدت یک بیماری یا تغییرات کلی وضعیت بیمار بعد از درمان را گزارش می کنند) ۳۳ .(	کمی / گسسته quantitative/ Discrete	وابسته Dependent	میزان تغییرات بعد از درمان از نگاه بیمار
-	نسخه فارسی پرسشنامه ICIQ-FLUTS LF	نشانه های ذهنی یک بیماری که توسط بیمار، مراقب یا خانواده اش درک میشود و را به سمت پیدا کردن درمان سوق میدهد. این دسته از نشانه ها معمولا در شرح حال گیری از بیمار به دست می آیند و معمولا کیفی هستند).۲۷(	کمی / پیوسته quantitative/continuous	زمینه ای Background	شدت علائم بی اختیاری ادراری
سال	پرسشنامه	سن افراد بصورت تقویمی و برحسب سال و ماه و روز سپری شده از زمان تولد آنها بیان میگردد	کمی / گسسته quantitative/ Discrete	زمینه ای Background	سن
کیلوگرم	ترازوی استاندارد	وزن بدن انسان عبارت است از مقدار جرم بدن یک فرد .	کمی / پیوسته quantitative/continuous	زمینه ای Background	وزن
سانتی متر	متر نواری	قد انسان عبارت است از فاصله کف پا تا بالای سر هر شخص در حالت ایستاده و عمودی.	کمی / پیوسته quantitative/continuous	زمینه ای Background	قد

هزینه پرسنلی

جمع	حق الزحمه در ساعت (ریال)	مجموع ساعات	نوع فعالیت	مرتبه علمی	نام مجري/همکار
۳۰'۰۰۰'۰۰۰	۳۰۰'۰۰۰	۱۰۰	ارزیابی بیماران	هیات علمی/استادیار	سیده سعیده بابازاده
۱۵'۰۰۰'۰۰۰	۱۵۰'۰۰۰	۱۰۰	درمان بیماران	غیر هیات علمی/دانشجو	فهیمه کارشناس
۲۷'۰۰۰'۰۰۰	۳۰۰'۰۰۰	۹۰	ارجاع بیمار	هیات علمی/استادیار	سنا طیبی
۲۲'۵۰۰'۰۰۰	۲۵۰'۰۰۰	۹۰	تجزیه و تحلیل داده ها	غیر هیات علمی/دکتری	نیلوفر ربیعی
۱۶'۰۰۰'۰۰۰	۴۰۰'۰۰۰	۴۰	ارجاع بیمار	هیات علمی/استاد	مریم امامی
۱۸'۰۰۰'۰۰۰	۳۰۰'۰۰۰	۶۰	تجزیه و تحلیل داده ها	هیات علمی/استادیار	سید محمد جعفر حائری
۱۲۸'۵۰۰'۰۰۰					**مجموع کل**

هزینه مسافرت

برای هزینه مسافرت هیچ داده ای ثبت نشده است.

هزینه های دیگر

عنوان هزینه	مبلغ هزینه (ریال)	جمع
چاپ و تکثیر پرسش نامه. به تعداد حجم نمونه و تعداد دفعات تکرار	۳۰’۰۰۰’۰۰۰	۳۰’۰۰۰’۰۰۰
**مجموع کل**	۳۰’۰۰۰’۰۰۰	

هزینه آزمایشات و خدمات تخصصی

موضوع آزمایش	نوع آزمایش	هزینه هر بار آزمایش (ریال)	محل آزمایش	تعداد آزمایش	جمع
یوروفلومتری	دستگاه و مواد Device and materials	۴’۵۰۰’۰۰۰	بیمارستان های دانشگاه علوم پزشکی ایران	۳۰	۱۳۵’۰۰۰’۰۰۰
تحریک الکتریکی	دستگاه Device	۴۰۰’۰۰۰	کلینیک فیزیوتراپی دانشکده علوم توانبخشی	۳۰۰	۱۲۰’۰۰۰’۰۰۰
سونوگرافی	دستگاه و مواد Device and materials	۱’۵۰۰’۰۰۰	کلینیک سونوگرافی	۳۰	۴۵’۰۰۰’۰۰۰
**مجموع کل**	۳۰۰’۰۰۰’۰۰۰				

هزینه وسایل و مواد خریداری شده

نام وسیله	مصرفی یا غیرمصرفی	تعداد یا مقدار	قیمت واحد (ریال)	شرکت سازنده	مقیاس	جمع
پد الکتروتراپی	مصرفیusage	۶۰	۷۰۰’۰۰۰	نهال	تعدادNumber	۴۲’۰۰۰’۰۰۰
**مجموع کل**	۴۲’۰۰۰’۰۰۰					

تامین اعتبار

نوع تامین اعتبار	نام موسسه/سازمان/مرکز/دانشکده تامین کننده اعتبار	نحوه پرداخت	مبلغ تامین اعتبار از سوی مرکز هدف دوم)ریال(	مبلغ تامین اعتبار(ریال) (خارج از دانشگاه یا مرکز مستقل)	جمع
مشترک داخل دانشگاه Common within the university	دانشگاه علوم پزشکی ایران		۰		۰
**مجموع کل**	۰				

ملاحظات اخلاقی

ملاحظات و مشکلات اخلاقی طرح

۱) گروه بندی و درمان متفاوت در هر گروه منجر به امکان تفاوت در پیامد درمان می شود.

راه حل مشکلات اخلاقی

۱) قبل از انجام مداخله توضیح کاملی از گروه بندی و درمان در هر گروه به همه بیماران داده خواهد شد و به همه بیماران این اطمینان داده خواهد شد که درصورت اثبات هرگونه بهبودی معنادار بالینی در هر گروه، بعد از اتمام زمان اجرای درمان ها، درمان مفید واقع شده، برای گروه دیگر نیز با همان طول مدت و کیفیت اجرا خواهد شد.
۲) بیماران برای ورود به طرح کاملا مختار هستند، لذا از همه بیماران رضایت نامه کتبی آگاهانه قبل از آغاز درمان گرفته خواهد شد.
۳) اصل رازداری و حفظ داده های جمع آوری شده و اطلاعات بیمار رعایت خواهد شد.
۴) به همه شرکت¬کنندگان در این مطالعه اختیار داده خواهد شد که در هر مرحله از مطالعه که تمایل به ادامه و همکاری نداشته باشند می توانند از مطالعه خارج شوند.

آیا طرح رضایتنامه اخلاقی دارد

بلی

اینجانب متعهد میشوم تا قبل از اخذ مصوبه و تاییدیه کمیته اخلاق دانشگاه طرح خود را شروع ننموده ام و چنانچه طرح اینجانب نیاز به اخذ رضایت آگاهانه داشته باشد متعهد می گردم هنگام ارایه اولین گزارش. نسبت به ارسال۱۰ درصد از فرم رضایتنامه تکمیل شده از بیماران به همراه شماره تماس و نام بیماران مورد مطالعه را ارسال کنم.

بلی

فرم رضایت نامه

۱.من می دانم که اهداف این پژوهش عبارتند از:

تعیین و مقایسه اثرات جریان اینترفرنشیال و درمان یوروتراپی روتین بر علائم بالینی و یافته های یورودینامیک در زنان مبتلا به اختلال تخلیه ادرار

۲.من می دانم شرکت من در این پژوهش کاملا داوطلبانه است و مجبور به شرکت در این پژوهش نیستم به من اطمینان داده شد که اگر حاضر به شرکت در این پژوهش نباشم، از مراقبت های معمول تشخیصی و درمانی محروم نخواهم شد و رابطه درمانی من بامرکز درمانی و پرشک معالج دچار اشکال نشود

۳.من می دانم که حتی پس از موافقت با شرکت در پژوهش می توانم هر وقت که بخواهم، پس از اطلاع به مجری، از پژوهش خارج شوم و خروج من از پژوهش باعث محرومیت از دریافت خدمات درمانی معمول برای من نخواهد شد.

۴.نحوه ی همکاری اینجانب در این پژوهش به این صورت است:

در جلسات درمانی برای دریافت درمان های روتین اختلالات ادراری شامل تمرین درمانی و تحریک الکتریکی واقعی و شم شرکت خواهم کرد. جلسات درمان برای هر دو گروه شامل ۱۰ جلسه درمان به صورت ۲ جلسه در هفته برگزار خواهد شد. جریان الکتریکی واقعی و شم هر کدام به مدت ۲۰ دقیقه در هر جلسه اعمال خواهد شد. همچنین مابقی جلسه درمان شامل یوروتراپی و تمرین درمانی می شود.

۵.منافع احتمالی شرکت اینجانب در این مطالعه به شرح زیر است:

بهبود در علایم اختلالات ادراری و بهبود کیفیت زندگی
مشارکت در ارتقا یافته های درمانی برای بیماران مبتلا به اختلالات ادراری

۶.آسیب ها و عوارض احتمالی شرکت در این مطالعه به این شرح است:

این مطالعه از تحریک الکتریکی تایید شده در درمان های فیزیوتراپی استفاده خواهد کرد بنابراین عوارض احتمالی بسیار محدود بوده و تنها میتواند به کمی قرمزی در محل الکترود محدود شود.
در صورتی که در هر یک از مراحل مطالعه پیش رو، علائم مرتبط با اختلال تخلیه ادرار در بیمار تشدید شد، بیمار سریعا جهت معاینه و بررسی های بیشتر به پزشک متخصص اورولوژی ارجاع داده خواهد شد.

۷.در صورت عدم تمایل به شرکت در مطالعه روش معمول درمانی برای من ارائه خواهد شد که منافع و عوارض آن به این شرح است:

استفاده از دارو درمانی برای اختلالات ادراری که شامل منافع و عوارض خاص استفاده از داروها می باشد

۸.من می دانم که دست اندرکاران این پژوهش، کلیه اطلاعات مربوط به من را نزد خود به صورت محرمانه نگه داشته و فقط اجازه دارند فقط نتایج کلی و گروهی این پژوهش را بدون ذکر نام و مشخصات اینجانب منتشر کنند.

۹.می دانم که کمیته اخلاق در پژوهش با هدف نظارت بر رعایت حقوق اینجانب می تواند به اطلاعات من دسترسی داشته باشید

۱۰.من می دانم که هیچ یک از هزینه های انجام مداخلات پژوهشی به شرح ذیل بر عهده من نخواهد بود.

هزینه دریافت درمان فیزیوتراپی (جریان اینترفرنشیال) برای اختلالات ادراری

---

۱۱ مشخصات فردی که جهت پاسخگویی به اینجانب معرفی شد و به من گفته شد تا هر وقت مشکلی یا سوالی در رابطه با شرکت در پژوهش مذکور پیش آمد با ایشان در میان بگذارم و راهنمایی بخواهم. نام و آدرس و شماره تلفن ثابت و همراه ایشان به شرح زیر به من ارائه شد.:

نام و نام خانوادگی

سیده سعیده بابازاده

آدرس:

تهران میرداماد میدان مادر خ شاه نظری خ مددکاران دانشکده توانبخحشی ایران

تلفن ثابت

۰۲۱۲۲۲۲۷۱۲۴

تلفن همراه:

۰۹۰۲۹۶۸۵۱۵۴

۱۲.من می دانم که اگر در حین و بعد از انجام پژوهش هر مشکلی اعم از جسمی و روحی به علت شرکت در این پژوهش برای من پیش آمد درمان عوارض آن و غرامت مربوطه بر عهده مجری خواهد بود.

۱۳.من می دانم اگر اشکال یا اعتراضی نسبت به دست اندرکاران یا روند پژوهش دارم میتوانم با کمیته اخلاق در پژوهش دانشگاه علوم پزشکی ایران به آدرس : تهران،دانشگاه علوم پزشکی ایران ، بزرگراه شهید همت غرب بین تقاطع شیخ فضل الله و شهید چمران ستاد مرکزی طبقه ۵ معاونت تحقیقات و فناوری با شماره ۸۶۷۰۲۵۳۰ تماس گرفته و مشکل خود را به صورت شفاهی یا کتبی مطرح نماییم.

۱۴.این فرم اطلاعات و رضایت آگاهانه در دو نسخه تهیه شده و پس از امضا یک نسخه در اختیار من و نسخه دیگر در اختیار مجری قرار خواهد گرفت.

ضمایم

نام فایل	نوع فایل	توضیح	زمان آپلود	دانلود
حکم کارگزینی ۱۴۰۳.pdf			۰۹/۰۲/۱۴۰۳ ۱۳:۱۲:۴۵	دانلود
نامه به دانشگاه.pdf			۰۹/۰۲/۱۴۰۳ ۱۳:۱۳:۲۳	دانلود
c.docx۶۵۳f۸f۱۶be۴a۹f۳۰۴۵۷۴۰۱۳۹d۵۱۱۱۱۷	سایر پیوست ها	رضایت نامه کتبی	۰۵/۰۶/۱۴۰۳ ۰۹:۵۳:۴۴	دانلود

مجموع کل هزینه ها

هزینه	مجموع (ریال)
مجموع هزینه های پرسنلی	۱۲۸'۵۰۰'۰۰۰
هزینه آزمایشات و خدمات تخصصی	۳۰۰'۰۰۰'۰۰۰
فهرست وسایل و مواد خریداری شده	۴۲'۰۰۰'۰۰۰
هزینه مسافرت	۰
هزینه های دیگر	۳۰'۰۰۰'۰۰۰
اعتبار از سایر سازمان ها	۰
**مجموع کل هزینه ها**	۵۰۰'۵۰۰'۰۰۰
بودجه تامین شده توسط بخش همکار داخلی (مرکز دوم)	۰
**کسر مبلغ اعتبار اختصاص یافته از سایر سازمان ها**	۰
**بودجه مورد نیاز (مجموع کل بدون اعتبار خارج از دانشگاه)**	۵۰۰'۵۰۰'۰۰۰
