# Supplementary material for: Effects of interferential stimulation on clinical symptom and urodynamic findings in women with voiding dysfunction: A protocol of randomized clinical trial
Source: PLoS One. 2025 Oct 27;20(10):e0330610. doi: 10.1371/journal.pone.0330610 (PMC12558555; doi:10.1371/journal.pone.0330610)
Supplement: Attachment — Submitted filename: Response to reviewers.docx [file pone.0330610.s004.docx]

Reviewer #1: The authors reported a study aiming to describe the effects of inferential stimulation on clinical symptom and urodynamic findings in women with voiding dysfunction. Generally the study is interesting, but it has some issues. Below there are some comments which need to be taken into account during the revision of this manuscript.

**We sincerely appreciate your valuable comments. All suggested revisions have been carefully incorporated and are highlighted in green for your reference**.

1. Please define voiding dysfunction according to the ICS guidelines.

-**Response**: We appreciate your thoughtful consideration. This definition has been incorporated into both the introduction (lines 60-63) and the Abstract (line 22) of the manuscript.

2. The VD is a general term. Which type of VD patients will include in the study? Describe it completely.

-**Response**: We appreciate your thoughtful review. The manuscript now includes a comprehensive description of dysfunctional voiding (lines 24-27 in Abstract, and lines 85-87 in main text), which has been systematically incorporated throughout the text where relevant.

3. Please define positive urodynamic criteria favor the voiding dysfunction diagnosis in the inclusion criteria part.

- **Response**: We appreciate your thoughtful consideration. This criterion has been incorporated into the eligibility criteria section in Lines 167-169: (Q max equal to or less than 12 milliliters per second, a detrusor pressure at the maximum urinary flow rate equal to or more than 20, and active EMG in voiding phase).

4. Please include these criteria as exclusion criteria “ 5) absence of pregnancy and breastfeeding, 6) no history of spinal and pelvic surgery in the last 2 years, 7) no history of back and pelvic malignancies in the last 2 years, 8) absence of structural disorder, neurological disorder, history of chronic disease [1], 9) absence of neurological bladder [1], 10) absence of voiding dysfunction secondary to drug use or other diseases [1], 11) absence of high degrees of pelvic organ prolapse (degrees higher than 2)”.

- **Response**: We sincerely appreciate your valuable feedback. The recommended criteria have now been incorporated into the exclusion criteria section (Lines 171-178), with all necessary textual revisions implemented.

5. Why do you will concern only on Qmax measurement in this study? What about other parameters of uroflowmetry such as voiding volume, Qave, voiding time ?

-**Response**: Thanks for your valuable recommendation. While patients with dysfunctional voiding typically maintain normal voided volumes unaffected by treatment, we fully acknowledge the value of comprehensive urodynamic assessment. While maximum urinary flow rate (Qmax) serves as the primary outcome, we appreciate this insightful comment and confirm that our assessment protocol includes multiple secondary outcome measures (Urinary flow patterns, average flow rate (Qave), micturition duration, voided volume, flow time, Pdet@Qmax) as detailed in the Outcome Measures section (Lines182-183, and 229-231).

6. What is the purpose of using these parameters” a carrier frequency of 4000 Hz and a beat frequency of 160-80 Hz” of IF therapy in this study? Which condition will be improved? Please add a reference.

- **Response**: We appreciate your thoughtful consideration. This protocol represents the most frequently employed and evidence-based approach for pelvic floor disorders, as established by consensus across studies investigating interferential current therapy - including those focusing on voiding dysfunction (The following references). we added the references to this sentence. line 338.

*-Sharifi-Rad L, Seyedian S-SL, Fatemi-Behbahani S-M, Lotfi B, Kajbafzadeh A-MJJoPU. Impact of transcutaneous interferential electrical stimulation for management of primary bladder neck dysfunction in children. 2020;16(1):36. e1-. e6.*

*-Sharifi-Rad L, Ladi-Seyedian S-S, Kajbafzadeh A-MJUJ. Interferential Electrical Stimulation Efficacy in the Management of Lower Urinary Tract Dysfunction in Children: A Review of the Literature. 2021;18(5).*

7. Will you use sham stimulation in the control group? If yes, why the output intensity will be adjusted to the patient's initial sensation threshold and the device output will be terminated after one minute? Is increasing of intensity in sham stimulation correct?Please clear it.

- **Response**: We appreciate your comment. Yes, sham stimulation will be used in the control group. To mitigate the placebo effect of interferential electrical current and selection bias, sham current and patient blinding to the group allocation will be implemented. Various methods have been employed in previous studies to address this purpose while using electrical currents. Recent studies, in addition to using identical electrode placement in both groups, have applied interferential current for a short period during treatment and then discontinued the current. In the present study, to blind patients to the type of current used interferential electrical current will be applied for the initial 1 minute of treatment in the control group. To minimize the therapeutic effects of this modality, the current intensity will be adjusted to the initial sensation threshold of patients in the control group; however, the treatment group will receive current at an intensity tolerable for the individual. According to literatures, interferential current should be applied for at least 20 minutes with proper intensity for effectiveness. So, exposure to this stimulation for this short period of time and this inappropriate intensity will cause no bias. lines 343 and 351.

*- Mendonça Araújo F, Alves Menezes M, Martins de Araújo A, Abner dos Santos Sousa T, Vasconcelos Lima L, Ádan Nunes Carvalho E, et al. Validation of a New Placebo Interferential Current Method: A New Placebo Method of Electrostimulation. Pain Medicine. 2017;18(1):86-94.*

*- Day SJ, Altman DG. Statistics notes: blinding in clinical trials and other studies. BMJ (Clinical research ed). 2000;321(7259):504.*

*- Rakel B, Cooper N, Adams HJ, Messer BR, Frey Law LA, Dannen DR, et al. A new transient sham TENS device allows for investigator blinding while delivering a true placebo treatment. The journal of pain. 2010;11(3):230-8.*

*- Watson T. Interferential Therapy (IFT). 2015.*

Reviewer #2: Statistically the manuscript is well designed and the analysis is simple. There are a few clarifications needed.

**We sincerely appreciate your valuable comments. All suggested revisions have been carefully incorporated and are highlighted in yellow for your reference**

1. The sample size section is incomplete?
In the sample size section, what effect size is being used for the intervention comparison? How did one arrive at 14 per group? In the sample size calculation method in the supplementary protocol there is even less information justifying 14 per group.

- **Response:** We appreciate your thoughtful consideration. In this study, the sample size of maximum urine flow (Qmax) as a primary outcome was calculated with G*Power 3.1.9.2 software. Twenty-eight subjects will be included in this trial, fourteen subjects in two of the groups (with an expected 10% dropout rate). α and power (1- β) values for the sample size calculation, using repeated measures analysis of variance (ANOVA) for three measurements in two groups, were set at 0.05 and 0.8, respectively. Additional parameters factored into the sample size calculation included the effect size, number of groups, number of measurements, and the correlation between repeated measures. The effect size was derived from a study conducted by Kajbafzadeh, which reported mean (standard deviation) Qmax values of 21 (8.3) in the intervention group and 12.8 (4.8) in the control group after a one-year follow-up . Based on these values, the calculated effect size was 0.854. The number of groups and measurements used in the analysis are two and three, respectively. Correlation values between repeated measures ranged from 0 to 0.99; ultimately, a correlation of 0.7 was selected, as it yielded the largest required sample size.

The complete implementation is detailed in lines 395-404 of the manuscript.

*Kajbafzadeh AM, Sharifi‐Rad L, Ladi‐Seyedian SS, Mozafarpour SJBi. Transcutaneous interferential electrical stimulation for the management of non‐neuropathic underactive bladder in children: a randomised clinical trial. 2016;117(5):793-800.*

2. In the Statistical analysis section, a Two way repeated measure ANOVA and one way ANOVA are mentioned. Why can’t one get the group (intervention) and time affects from the repeated measures ANOVA and test for interaction as well?

- **Response:** We appreciate your thoughtful comment. To evaluate the effects of time (before the intervention, after the intervention, and after a three-month follow-up) on measurement outcomes—while considering group as a between-subject factor—repeated measures ANOVAs will be employed, assuming the data are normally distributed. In cases of non-normality, a nonparametric equivalent will be applied. Both a within-subject factor (Time) and a between-subject factor (Group) will be included in the analysis. Interaction effects (Time × Group) will be examined to determine whether group differences influenced changes in measurements over time.

To assess the effects of time on measurement outcomes within each group, separate one-way repeated measures ANOVAs will be conducted—one for the treatment group and one for the control group. If a statistically significant difference in the mean values over time is found, post hoc tests with Bonferroni and Tukey corrections will be used.

The complete implementation is detailed in lines 414-426 of the manuscript.

3. Unless I am misunderstanding the primary endpoints, it appears that there are about three primary outcome measures (urinary flow patterns, maximum urinary flow rate, and duration of urination). There may actually be four as per the supplementary protocol. What adjustment is being made for multiple comparisons? This will affect the overall type I error.

- **Response:** This study has two primary outcomes: maximum urine flow (Qmax) and severity of lower urinary tract symptoms. To maintain clarity, we’ve consistently specified Qmax as the primary outcome measure wherever relevant in the manuscript and have categorized other uroflowmetry parameters in the secondary outcome measures section.

lines: 191, and 222.

During the analysis process, each outcome will be treated as an independent response, and analyses such as two-way repeated measures analysis, one-way repeated measures analysis, and independent samples t-tests will be employed. In the case of multiple comparisons, as previously noted in the statistical analysis section, Tukey and Bonferroni post hoc tests (designed to maintain the overall Type I error rate at or below a desired threshold—typically 0.05) will be applied.

4. The document is nicely written in general. There are a few typos. Please check for typos:
e.g. line 415 , in the ‘Adverse’ Events section, “Also, it is easy to application”. Should be, “Also, it is easy to apply.”
Also, the sentence structure may be awkward in a number of places. Thanks!

-**Response**: Thank you for your comments. The text has been revised again, and typos and grammatical corrections have been made. lines 491-492.

Reviewer #3: Thank you for submitting this protocol. I don't have any major concerns about the grammar or flow of the manuscript - before resubmitting, check the capitalization of your affiliations. There are a few areas that need a bit more detail to help with my understanding:

**We sincerely appreciate your valuable comments. All suggested revisions have been carefully incorporated and are highlighted in blue for your reference.**

- Thank you so much. Capitalization of affiliations were corrected. line 11

1. Under Study Design (line 141), it states that "Patients will be recruited through urologist referrals". However, under Recruitment (line 146-47), it states that "Sampling will be conducted using a non-probability convenience sampling
method, based on specialist referrals and public announcements on social media." Please clarify how participants are being recruited, so it is consistent between the two sections.

- **Response**: Thank you for your comment. Yes, that is correct. These two sections have been clarified and patient referrals will only be made through a specialist and according to the inclusion criteria. The revised methodology detailing this standardized referral protocol appears in the Recruitment subsection (Line 151).

2. Outcomes section
a. I recommend including the minimum clinically important difference (MCID) for the ICIQ-FLUTS, PFDI-20, and GRCS. These values will help to determine if the changes noted with the intervention are clinically meaningful.

-**Response**: We thank the reviewer for this helpful comment. In response, we have incorporated the minimum clinically important differences (MCIDs) for all assessment tools, with detailed justification provided in the statistical analysis section (Lines 433-448).

*ICIQ-FLUTS: We cited Islam et al. (2023), who reported an anchor-based CID of approximately 3.4 points and MID values ranging from 1.3 to 3.2, depending on treatment type and duration. Although their population consisted of women with stress incontinence, we applied their thresholds with appropriate caution, given the overlapping voiding domains relevant to our sample. PFDI-20: We acknowledged the MCID of 45 points reported by Barber et al. (2005) in a surgical cohort. Although our population received non-surgical treatment, this remains the most widely accepted MCID in the literature. We highlighted this discrepancy in the revised manuscript to promote interpretive transparency. GRCS: The GRCS was not used as an outcome measure, but rather as an anchor to estimate MCID for the ICIQ-FLUTS and PFDI-20, following the original methodology proposed by Jaeschke et al. (1989). We defined* *“a little better” on the GRCS as the threshold for minimal important change. Additionally, where appropriate, we will estimate our own anchor-based MCID values within our sample using GRCS response categories and will include these findings in the Results section for completeness.*

*-Nipa SI, Cooper D, Mostafa A, Hagen S, Abdel-Fattah M. Novel clinically meaningful scores for the ICIQ-UI-SF and ICIQ-FLUTS questionnaires in women with stress incontinence. International urogynecology journal. 2023;34(12):3033-40.*

*-Barber MD, Walters MD, Bump RC. Short forms of two condition-specific quality-of-life questionnaires for women with pelvic floor disorders (PFDI-20 and PFIQ-7). American journal of obstetrics and gynecology. 2005;193(1):103-13.*

*-Kamper SJ, Maher CG, Mackay G. Global rating of change scales: a review of strengths and weaknesses and considerations for design. The Journal of manual & manipulative therapy. 2009;17(3):163-70.*

b. Please provide a bit more detail about how the bladder diary will be completed. For example, how will participants measure voiding volumes and fluid intake? Which three days will be selected to complete the bladder diary (e.g., weekdays versus weekends)? What will be done to help with fully completing the bladder diaries (I often find patients have difficulties filling them out for the full three days)?

-**Response**: We appreciate your thoughtful consideration. A full explanation of how to fill out the diary has been added to the text. Lines 253-278.

3. Treatment protocol section
a. Please provide more details about how the physiotherapist will guide pelvic floor contractions (lines 275-277), will they be using ultrasound, external palpation, or an internal exam?

-**Response**: The physiotherapist will initially assess proper pelvic floor contractions through visual observation of superior perineal movement during the first instructional session. Subsequent verification will be conducted via external palpation when visual assessment proves insufficient to confirm correct muscle engagement. These sentences have been added to lines 317-321.

b. Please clarify the pelvic floor muscle training parameters - is the participant expected to to one set of 10 in each of the three positions?

-**Response**: We appreciate your thoughtful comment. The treatment protocol follows a progressive weekly schedule, with each exercise position specifically assigned to a particular treatment week. Patients are instructed in one new position per week, beginning with the supine position in week one and progressing to functional positions in subsequent weeks. The complete positional sequence and corresponding weekly schedule are detailed in Table 1.

c. Will the control group remain connected to the IFC for the full 20 minutes, but with the current disconnected after 1 minute? Please clarify in lines 303-307 if the duration attached to the machine will be the same as the treatment group.

-**Response**: Yes, due to the performing complete process of blinging, the device will remain attached to sham group participants for the full 20-minute session duration despite delivering no active stimulation, maintaining consistent experimental conditions across groups*.* This explanation added in lines 352-355.

4. Statistical analysis
a. To make it clear to the reader, please list the demographic quantitative variables and the qualitative variables in the first paragraph of this section.

- **Response**: The requested information has been added to the main text as follows in lines 406-407: Demographic characteristics (age and BMI) will be summarized using descriptive statistics, presented as mean ± standard deviation for both study groups*.*

5. References
a. I'd recommend referring to the most recent publications as possible (e.g., Incontinence 7th Edition - <https://www.ics.org/education/icspublications/icibooks>) when developing your inclusion criteria and describing the urodynamic testing you'll be using.

-**Response**: Thank you for your recommendation. We have added some references from ICS to manuscript. References number 1 and 19.
