# Supplementary material for: Effects of interferential stimulation on clinical symptom and urodynamic findings in women with voiding dysfunction: A protocol of randomized clinical trial
Source: PLoS One. 2025 Oct 27;20(10):e0330610. doi: 10.1371/journal.pone.0330610 (PMC12558555; doi:10.1371/journal.pone.0330610)
Supplement: Attachment — Submitted filename: response to reviewers and editor.docx [file pone.0330610.s005.docx]

I would like to express my sincere gratitude to the journal’s editor and all reviewers for their careful review.

All requested corrections have been addressed, and responses to specific questions are provided below.

**Response to editor:**

1.Ensure the accurate use of the terms "dysfunctional voiding" and "voiding dysfunction". As noted by the first reviewer, the term "dysfunctional voiding" is primarily a pediatric diagnosis. In older patients, alternative terms such as "voiding dysfunction", "non-neurogenic detrusor sphincter dyssynergia", or "functional bladder outlet obstruction" may be more appropriate. Please ensure uniformity in definitions and terminology throughout.

- **Response**: Regarding voiding dysfunction (as noted in the manuscript), this term broadly encompasses impaired detrusor contractility or bladder outlet obstruction (BOO). In women, BOO may arise from structural or functional causes, with major functional causes including dysfunctional voiding and detrusor-sphincter dyssynergia. Dysfunctional voiding is characterized by involuntary periurethral striated muscle contractions during voiding in neurologically normal individuals, resulting in an intermittent and/or fluctuating urine flow rate.

While the ICS definition of dysfunctional voiding does not specify age, we agree with the first referee’s observation that it occurs more frequently in younger individuals. Consequently, we narrowed the inclusion criteria’s age range to premenopausal women.

2. We also recommend a final round of proofreading for grammatical and typographical accuracy. A thorough English language edit by a native speaker, as well as a formatting and layout edit would be appreciated. Additionally, kindly eliminate redundancies where possible.

-	**Response**: The manuscript was revised for typo, punctuation, grammer, and spelling. errors.

3.In the statistical analysis section, several tests are described more than once and could be consolidated,

-	**Response**: We would like to clarify that repeated measures ANOVA was intentionally used for two different analytical goals in our study and it is the reason of the repetition in this section:

First, to evaluate the interaction between time and group, we applied a repeated measures ANOVA including both study groups in the same model. This enabled us to assess whether the treatment group differed from the control group in how their outcomes changed over time.

Second, to assess within-group changes over time, repeated measures ANOVAs were conducted separately for each study group. This allows us to explore how the outcomes evolved independently in the treatment and control groups without the influence of inter-group comparisons.

We appreciate your feedback and have revised the Statistical Analysis section to consolidate overlapping descriptions and improve clarity.

4. In the inclusion and exclusion criteria section, the reference numbers are intermingled with the actual criteria causing confusion. You may consider placing the references at the beginning of the section or alongside the first mention of the criteria.

-	**Response**: The references in the inclusion criteria section were revised and -relocated to the end of section

5. Some subheadings such as "Objectives" (line 135), and "Participants Schedule" (line 165) may be removed to streamline the study.

-	**Response**: these subheadings were removed.

**Response to reviewers**

Reviewer #1: I would like to thank the authors who made the necessary corrections to develop the manuscript. Only I have one question.
is it permissible to use the term of dysfunctional voiding in older women? Because pelvic floor muscle weakness or bladder neck disorders are more common in these older women, and the diagnosis of dysfunctional voiding was made by positive EMG activity during voiding..

- **Response**: Regarding voiding dysfunction (as noted in the manuscript), this term broadly encompasses impaired detrusor contractility or bladder outlet obstruction (BOO). In women, BOO may arise from structural or functional causes, with major functional causes including dysfunctional voiding and detrusor-sphincter dyssynergia. Dysfunctional voiding is characterized by involuntary periurethral striated muscle contractions during voiding in neurologically normal individuals, resulting in an intermittent and/or fluctuating urine flow rate.

While the ICS definition of dysfunctional voiding does not specify age, we agree with your observation that it occurs more frequently in younger individuals. Consequently, **we narrowed the inclusion criteria’s age range to premenopausal women.**

Reviewer #2: None
XXXXXXXXXXXXXXXXXXXXXXXXXXXXXXXXXXXXXXXXXXXXXXXXXXXXXXXXXXXXXXXXXXXXXXXXXXXXXXXXXXXXXXXXXXXXXXXXXXXXXXXXXXXXXXXXXXXXXXXXXXXXXXXXXXXXXXXX

Reviewer #3: Thank you for making changes to the manuscript, addressing our concerns. I noticed some small typos, missing punctuation, and grammatical errors during my review - I'd encourage you to ask a friend or colleague not associated with this manuscript to proof read the final submission.

- **Response**: The manuscript was revised for typographical errors, punctuation, and spelling was corrected
